# Exploring the latent space of transcriptomic data with topic modeling

**DOI:** 10.1093/nargab/lqaf049

**Published:** 2025-04-22

**Authors:** Filippo Valle, Michele Caselle, Matteo Osella

**Affiliations:** Physics Department, University of Turin and INFN, Via Pietro Giuria 1, 12125 Torino, Italy; Physics Department, University of Turin and INFN, Via Pietro Giuria 1, 12125 Torino, Italy; Physics Department, University of Turin and INFN, Via Pietro Giuria 1, 12125 Torino, Italy

## Abstract

The availability of high-dimensional transcriptomic datasets is increasing at a tremendous pace, together with the need for suitable computational tools. Clustering and dimensionality reduction methods are popular go-to methods to identify basic structures in these datasets. At the same time, different topic modeling techniques have been developed to organize the deluge of available data of natural language using their latent topical structure. This paper leverages the statistical analogies between text and transcriptomic datasets to compare different topic modeling methods when applied to gene expression data. Specifically, we test their accuracy in the specific task of discovering and reconstructing the tissue structure of the human transcriptome and distinguishing healthy from cancerous tissues. We examine the properties of the latent space recovered by different methods, highlight their differences, and their pros and cons across different tasks. We focus in particular on how different statistical priors can affect the results and their interpretability. Finally, we show that the latent topic space can be a useful low-dimensional embedding space, where a basic neural network classifier can annotate transcriptomic profiles with high accuracy.

## Introduction

The number of large-scale and high-dimensional datasets of gene expression obtained with RNA sequencing is growing at a frantic pace [1, 2]. This data accumulation generates a parallel growing need for computational methods to identify the hidden relevant structures [3, 4]. For example, the efficient identification of patterns in expression data is a key step in the development of a precision medicine program, ranging from the discovery of disease-specific expression profiles for diagnosis to the stratification of patients [5]. Analogously, in the context of single-cell RNA sequencing data, one often needs to identify the different cell types and their expression hallmarks, and this hierarchical structure of cell types has to be inferred in the presence of several confounding factors [6].

For all these tasks, the number of different algorithms and tools that have been developed and applied to RNA sequencing data is huge [7]. For example, clustering algorithms are classic go-to methods to find groups at the level of samples (e.g. patients or single-cells). At the gene level, clustering can instead be used to extract the genes whose expression is collectively orchestrated in a particular system or in response to treatment.

Dimensionality reduction is another key step in many standard computational pipelines. For example, Principal Component Analysis or analogous nonlinear methods, such as t-SNE, are widely used for both data-preprocessing and visualization [5, 8, 9]. The underlying hypothesis is that the number of relevant variables needed to efficiently describe the system is well below the number of genes, and thus concise but informative representations can be built [10].

This paper analyzes a class of algorithms, called topic models, that can essentially provide fuzzy clustering and dimensionality reduction simultaneously in a probabilistic framework [11]. Topic models were developed to organize texts of natural language using a space of latent variables, i.e. the topics, that define the different word usage probabilities, and can be interpreted as a more compact, low dimensional description of the dataset. Topics can be used to define a graded membership structure, or a fuzzy clustering, of texts in a corpus. While originally developed for natural language processing, topic modeling is recently finding applications in biology, and more specifically in genomics [3, 12–20]. Indeed, genomic data share several fundamental statistical laws with natural language and other modular complex systems [21–23].

However, the topic structure can be inferred using different methods that are based on alternative assumptions and priors. It is not clear a priori what are the pros and cons of each method, in particular in the genomic context. This is our focus in this paper. We will examine different approaches on simple and clear-cut benchmark datasets from RNA sequencing experiments. We will compare the performances of different algorithms in terms of clustering of samples and genes and analyze how their specific statistical priors affect the results in the context of genomic data. We will also discuss in detail the interpretability and biological relevance of the latent variables extracted by different topic modeling techniques.

The most popular topic modeling method is the Latent Dirichlet Allocation (LDA) [11]. It assumes that documents are generated from a mixture of topics, and each topic is characterized by a distribution over words. Iterative algorithms are used to infer the underlying topics and their distributions within the corpus. LDA employs Dirichlet priors to model the distribution of topics within documents and the distribution of words within topics. Such priors force few words to be representative of a topic, and analogously few topics to characterize each document. This choice is mostly motivated by mathematical and computational convenience, but it is not clear how appropriate it is in the genomic context, and what are its consequences. LDA has been previously applied to RNA sequencing data for data exploration [3], successfully retrieving genes whose expression can distinguish samples from different tissues.

Topic modeling has been recently connected to the problem of community detection in networks [24], and a new algorithm based on the hierarchical Stochastic Block Model (hSBM) has been formalized with the ambition to solve some of the weaknesses of LDA [25]. We have recently shown how this method can be used in the context of cancer genomics [16, 17], but a clear comparison with other topic modeling techniques in different genomic contexts is still needed.

Besides hSBM and LDA, we will also consider an alternative topic modeling approach called Topic Mapping (TM) [26], which has not been tested so far in the genomic context. This method uses the co-occurrence of words to create a network of tokens. Topics are then defined as basic network structures.

Topic modeling techniques will also be compared to more classic and popular computational methods such as the Weighted Gene Correlation Network Analysis (WGCNA) [27]. WCGNA is not formally a topic modeling method but still has several commonalities with this class of methods. In fact, WGCNA is widely used to search for correlated groups of genes, which are called gene ‘modules’, that can be loosely associated to topics. WGCNA does not provide a natural way to define clusters or groups of samples, but standard clustering techniques can be applied using the modules (i.e. the topics) as features.

Finally, we will also consider standard clustering methods such as the well-known hierarchical clustering algorithm [28]. With hierarchical clustering, we can search for groups of samples based on their relative Euclidean distance in the feature space. We can thus compare these groups to the clusters that different topic modeling methods identify. This comparison will provide a better understanding of the advantages of complex topic inference with respect to easy-to-interpret classic methods.

We will show that different algorithms generally find different topic distributions (or embedding spaces) with alternative structures and statistical properties. In general, there is not an optimal algorithm for every task [29]. Our goal is to highlight the potential biases and tendencies that affect different algorithms. In fact, even in presence of comparable performances on a specific task, different approaches can identify alternative structures and show different biases [30]. This finding represents an issue that has to be considered in the context of interpretability and causal inference. Interestingly, the topics we find with alternative methods, although often poorly overlapping, are generally well correlated with the biological properties of the samples analyzed.

Topic models also provide a low-dimensional space in which the samples can be represented. A simple neural network (NN) can be trained on this low-dimensional topic space with the task of labelling new samples. Specifically, a classifier in this space can distinguish samples from different tissues, as well as detect diseased samples over healthy ones with very high accuracy despite the dimensionality reduction.

Deep learning methods have also been applied in the context of transcriptomics. In particular, variational autoencoders are NNs that aim to infer the latent structure hidden in the data and can capture complex nonlinear relationships [31, 32]. However, these models typically have a large number of trainable parameters and several hyperparameters. This makes them highly flexible but also prone to overfitting and challenging to interpret. A thorough comparison of topic models and deep learning techniques for transcriptomics is a promising avenue for future analysis.

## Materials and methods

### The Genotype Tissue Expression dataset

The Genotype-Tissue Expression (GTEx) Project was supported by the Common Fund of the Office of the Director of the National Institutes of Health, and by NCI, NHGRI, NHLBI, NIDA, NIMH, and NINDS. The data used for the analyses described in this manuscript were obtained from the GTEx Portal phs000424.v8.p2 [33]. GTEx data were downloaded in Transcripts Per Million (TPM) format.

We also downloaded from the GTEx portal https://gtexportal.org the annotations of samples and in particular we focused on the tissue type (the area from which the tissue sample was taken) and its subtypes (SMTS and SMTSD labels).

We selected 1000 samples from each one of the 10 most represented (in terms of number of samples per tissue) tissues: Adipose tissue, Blood, Blood Vessel, Brain, Colon, Esophagus, Hearth, Muscle, Skin, and Thyroid.

### The mouse cell atlas

The Mouse Cell Atlas (MCA) database cointains single-cell transcriptomic data for major mouse organs. We considered data related to the five most represented cell-types from four different organs: Cerebellum, Heart, Liver, Lung. We subsample 100 cell of each cell-type per each Organ. Data with removed batch effects from the version 1.1 of the Atlas were downloaded from Figshare [34].

### Algorithms

In the following section we will describe the models and report the specific setting used for each method.

#### hierarchical stochastic block model

We used the stochastic block modeling topic modeling sbmtm class in the code we forked from [25] available at https://github.com/martingerlach/hSBM_Topicmodel/tree/develop. We passed the gene expression matrix as a weighted graph to the model.

The hSBM is a kind of generative model that tries to maximize the probability that the model describes the data


\begin{eqnarray*}
P(\theta | \mathcal {A}) \propto P(\mathcal {A} | \theta )P(\theta ).
\end{eqnarray*}

Its approach is completely nonparametric and aims to maximize the posterior probability. We used the minimise_nested_blockmodel_dl (the degree corrected version of the model was used) function from graph-tool [35] to minimize the description length $\Sigma = - ln P(\mathcal {A}|\theta ) - ln P(\theta )$ in a nested version of the model [36–40]. Minimizing Σ leads to the maximization of the posterior probability. In our setting $\mathcal {A}$ is nothing but the gene expression matrix, weighted by the number of *TPM* and θ refers to the model parameters. It is a 2D matrix with entries $\mathcal {A}_{ij}$: $\mathcal {A}_{ij}$ is the expression value of gene *i* in sample *j*. In a setting where rows are words and columns are documents it is often referred to as ‘Bag of Words’. The model is nonparametric, therefore the default version does not require the specification of hyperparameters. However, the model is intrinsically stochastic, thus we ran it 10 times for each setting and we chose the configuration with the shortest description length (i.e. the model with the best data fit).

#### Latent Dirichlet Allocation

When running analyses with LDA, we adopted the implementation provided by scikit-learn [41, 42]. LDA has few hyperparameters that have to be set, specifically the number of extracted topics *K*, and the sampling parameters α and β. We set the parameter *K* to match the number of topics obtained from hSBM (where the topic number is automatically set by hSBM itself). We used the natural default value for the parameters α (the parameter of the Dirichlet prior distribution of topics in documents) and β (the parameter of the Dirichlet prior of words in topics), which corresponds to $\frac{1}{K}$. When managing LDA output, we selected the *argmax* of *P*(topic|sample) to define clusters. Note that it is not guaranteed that every topic is maximized in at least one of the samples, so the number of clusters can be smaller than the number of topics. Using this procedure it is not possible to select a-priori the number of clusters. A similar procedure was used to identify clusters with TM.

To get a list out of the LDA topic distribution we selected the 20 most distinctive genes of each topic. The distinctiveness was described and used in LDA analyses of GTEx by [3] and is the minimum Kullback–Leibler distance of *P*(topic|gene).


(1) \begin{eqnarray*}
D^g[K]=min_{l\ne k} \theta _{kg} \log \left(\frac{\theta _{kg}}{\theta _{lg}}\right)+\theta _{lg}-\theta _{kg}
\end{eqnarray*}

being θ_{*k*, *l*}*g*_ the *P*(gene_*g*_|topic_{*k*, *l*}_).

#### Topic Mapping

We ran TM [26], with its default parameters: the number of runs -r was set to 10 and the minimum topic size -t was set to 10 to avoid small useless clusters and force the algorithm to put at least 10 samples per cluster.

TM requires as input a corpus of text and not a Bag of Words or a design matrix, this is why we built a corpus in which each text was constructed from the 1000 most expressed genes in each sample. In other words, each sample was translated into a text composed of the 1000 genes most represented in that sample.

Clusters are defined using the *argmax* of *P*(topic|sample).

Finally, we got the list of genes from the output of TM setting a threshold on *P*(topic|gene) corresponding to the 99th percentile and kept all the genes above that threshold.

#### Weighted gene correlation network analysis

We considered the R implementation by https://cran.r-project.org/web/packages/WGCNA/. WGCNA automatically outputs sets of genes (the topics) and it automatically selects their number. We considered WGCNA [27, 43] modules as topics, in particular, we chose the threshold of 0.5 to set the correlation for which genes are accepted in the module. The last step of WGCNA is to cut the hierarchy tree in order to obtain clusters, in this case, we cut it in a manner useful to obtain the same number of clusters in output by hSBM.

#### Hierarchical clustering

We use the agglomerative clustering class implemented in *sklearn* to perform hierarchical clustering. Agglomerative clustering has no hyperparameters, but one has to decide which metrics to consider to measure distances (we chose *euclidean* affinity) and how to group samples in clusters at the next level (we chose *complete* linkage), these choices were mutuated from previous works [3]. We would like to recall that our purpose is to use as little *a-priori* knowledge as possible, that is why, under reasonable assumptions, we chose standard settings without grid search hyper-parameters or applying other optimization methods. hSBM provides all the information we discussed without any a-priori assumption or parameter.

The input for the various algorithms was a matrix $\mathcal {A}$ whose entries are the gene expressions. Data of this matrix are samples and features are genes. In literature, this is often called ‘Bag of Words’.

One of the advantages of hSBM is that it automatically outputs the number of clusters and the clusters themselves. Hierarchical and WGCNA output a tree of samples; we need to cut it to obtain clusters. In LDA the number of topics is a parameter and in TM we set the minimal size of topics.

#### Computational complexity

hSBM was run on a node of a cluster [44] with 48 cores and 700 GB of memory and it took several hours to be run, all the other algorithms take minutes on a laptop with 2 cores and 8 GB of memory. The complexity of hSBM is *O*(*VLn*^2^*V*) only in the case of sparse networks when *E* ∼ *O*(*V*) [36], LDA has a complexity of *O*(K*N^2^) where *N* is the number of genes, *V* the number of vertices (samples and genes), *E* the number of edges, and *K* the number of topics. In our setting we have *V* = 1000 + 3000 = 4000 vertices, *E* ∼ 450 000 edges and tens of topics *K*. In this setting (i.e. dense network with $\frac{E}{V} \sim 10^2$) the complexity of hSBM is higher than the complexity of LDA.

### Model evaluation

We evaluated the performance in clustering by looking at the partition of samples between clusters. We used the V-measure or Normalized Mutual Information (NMI) [45] to evaluate the performance of the model in the task of separating samples of different tissues. A similar approach involving NMI was proposed by [46] to evaluate topic modeling performance in reconstructing synthetic corpora. NMI is estimated as the harmonic average of homogeneity and completeness. Homogeneity is defined as $h = 1-\frac{H(C|K)}{H(C)}$ and completeness is ${C} = 1-\frac{H(K|C)}{H(K)}$, so $NMI=2\frac{h*{C}}{h+{C}}$. In our setting *C* is the ensemble of GTEx tissues and *K* is the partition in output. In particular $H(C|K) = -\Sigma _{c \in C, k \in K}\frac{n_{ck}}{N}\log \left(\frac{n_{ck}}{n_k}\right)$ and $H(K|C) = -\Sigma _{c \in C, k \in K}\frac{n_{ck}}{N}\log \left(\frac{n_{ck}}{n_c}\right)$, being *n*_*c*_ the number of samples of tissue *c*, *n*_*k*_ the number of samples in cluster *k* and *n*_*ck*_ the number of samples of tissue *c* in cluster *k*. If all the nodes in cluster *k* belong to tissue *cn*_*k*_ = *n*_*ck*_ and the homogeneity *h* = 1. Similarly, the completeness *C* equals 1 if all samples of tissue *c* are in the same cluster *k*. See Supplementary Table S1 in the Supplementary for a brief illustration of these metrics.

This decomposition of the NMI can aid in understanding the biological implications of the clustering structure identified by the models. At the upper levels of the hierarchy, where the number of clusters is low, high completeness and low homogeneity indicate that clusters typically contain samples from multiple tissues (Supplementary Table S1). At this stage, the algorithms capture macro structures encompassing several tissues, which correspond to the known ground-truth partition in our illustrative example. As the hierarchy progresses and eventually aligns with tissue-level resolution, homogeneity increases and we often observe the NMI peak. In the deeper layers, where the number of clusters is higher, homogeneity reaches its maximum while completeness declines. Here, the algorithms focus on finer substructures within tissues (see Supplementary Table S1, lower-left panel). These trends suggest the existence of a true hierarchical structure within the data as the are not found with random partitioning at different scales. In the random case, both metrics only increase slightly after the initial trivial partitioning (see Supplementary Fig. S1).

We estimated the NMI score for all algorithms varying the resolution (layer). The NMI is not zero in the case of random sampling: when the number of clusters is too high the result is almost random for every algorithm. Therefore, we decided to normalize the NMI score with the one obtained by random partitions (i.e. we shuffled the data maintaining the number of clusters and the clusters sizes). The NMI for the random model was estimated for the number of clusters in output by hSBM, other points are interpolated from this. We referred to the NMI scaled to random as NMI*.

The random partitioning has an intrinsic variability in the NMI that induces variability in the NMI* scores associated to the different models. We can thus estimate score distributions and compare them. For example, Supplementary Fig. S2 reports the NMI* distribution obtained using multiple random partitions for the normalization in the case of transcriptomic data from different tissues.

We applied this approach to TPM data and on transformations of them. In particular, we applied two different log-transformations. We estimated log_*b*_(*TPM*_*ij*_ + 1) being *TPM*_*ij*_ the number of TPM of gene *i* in sample *j*. We tried with base *b* = 2 and *b* = 10 obtaining the same results.

### Hyper-geometric test and model comparison

Results of different algorithms were compared using two different approaches.

First, given the list of genes in two topics of different algorithms, we performed an hyper-geometric test. The test consists in evaluating the probability that the overlap between the two sets of happens by chance


(2) \begin{eqnarray*}
P(k)=\frac{\binom{K}{k} \binom{N-K}{n-k}}{\binom{N}{n}}
\end{eqnarray*}

where *N*, the population size, is the number of genes processed by both algorithms. *K*, the number of successes in the population, is the number of genes in the topic of the first algorithm. *n*, the sample size, is the number of genes in the topic of the second algorithm. The number of successes *k* is the intersection between the two sets i.e. the number of genes in common between the topics obtained by the two algorithms.

Note that some algorithms drop genes during the analyses. WGCNA has the *goodSamplesGenes()* function that returns a list of samples and genes that pass some criteria: missing entries, entries with weights below a threshold, and zero-variance genes. When we built corpora for TM it was not guaranteed that every gene was picked up at least once, and the same for the LDA topic-construction procedure, some genes can be left out.

The second approach is based again on the NMI. We used the same approach to compare clusters on the gene side and on the sample side. Given a partition in clusters of genes or samples the NMI was estimated as


\begin{eqnarray*}
NMI(X,Y)=\frac{I(X,Y)}{H(X)}=\frac{-\Sigma _{x,y}P(x,y)\log \left(\frac{P(x,y)}{P(x)P(y)}\right)}{-\Sigma _x P(x)\log \left(P(x)\right)},
\end{eqnarray*}

being *X* the set of cluster annotations (i.e. if *X* = {*c*_1_, *c*_1_, *c*_1_, *c*_2_…*c*_*k*_} it means that the first three samples belong to cluster *c*_1_...). *P*(*x*, *y*) is the probability that a sample is classified in cluster *x* by the first algorithm and in cluster *y* by the second algorithm. *P*(*x*) is the probability that a sample belongs to cluster *x*, namely the size of cluster *x* divided by the number of samples. NMI reaches one if the two partitions are identical: one gains no information reading the second sequence when the first is known. Notice that this measure is not affected by a reshuffling of cluster labels which are completely arbitrary.

### Gene selections

We used three different gene selection protocols.

#### Highly variable genes

this selection was performed using *scanpy* python package [47]. The package performs the analysis using the dispersion (variance over mean) [48]. Note that this kind of selection is always performed on the logarithmic data. See Supplementary Fig. S4 in Supplementary for an example of this kind of selection.

#### Housekeeping genes

These are typically constitutive genes that are required for the maintenance of basic cellular function. They are expressed in all cells of an organism under normal and pathophysiological conditions. We downloaded the list of 6289 housekeeping genes provided by [49]. Among these, we filtered a total of 3000 genes highly variable genes. The highly variable filter was performed taking into account only the housekeeping genes.

#### Random genes

These were selected using

the sample(random_state=42) function provided by the *DataFrame* module of *pandas* package.

### Topic distributions and fuzzy memberships

We are interested in studying the relevance of different topics in a given sample (or in a group of samples). To this end, we introduced a centered version of the *P*(topic|sample) distribution. Let us first recall the definition of *P*(topic|sample) in hSBM and LDA. In hSBM it is estimated as


(3) \begin{eqnarray*}
&&P(\text{topic}|\text{sample})=\nonumber\\ &&\frac{\text{number of half-edges on sample coming from topic}}{\text{number of half-edges on sample}}.\,
\end{eqnarray*}

In LDA it is a *K* dimensional vector θ_*i* = 1…*R*_ over topics (where *K* is the number of topics and *R* the number of samples), whose prior is a Dirichlet distribution θ_*i*_ = *Dir*(α) [50].

Topic models also allow to estimate the *P*(gene|topic) distributions that quantify the contribution of each gene to a topic. In hSBM, this is estimated as:


(4) \begin{eqnarray*}
&&P(\text{gene}|\text{topic})=\nonumber\\ &&\frac{\text{number of half-edges on gene going to topic}}{\text{number of half-edges going to topic}}.
\end{eqnarray*}

In LDA it is a *N* (number of genes) dimensional vector ϕ_*k* = 1…*K*_ over genes, whose prior is a Dirichlet distribution ϕ_*k*_ = *Dir*(β).

### Box topic and gene ontologies

Starting from *P*(topic|sample) we constructed a centered versions defined as:


(5) \begin{eqnarray*}
&&\overline{P}(\text{topic} | \text{sample}) =\nonumber\\ &&P(\text{topic} | \text{sample}) - \frac{1}{R}\Sigma _{s \in \text{samples}} P(\text{topic} | \text{s}),
\end{eqnarray*}

being *R* the total number of samples. This centered $\overline{P}(\text{topic} | \text{sample})$ can be represented with a box plot, after grouping samples by their tissue. These are the box plots reported in Fig. 7.

Topics are nothing but lists of genes, which are naturally brought to perform gene ontology tests. We searched for enrichment using Gene Set Enrichment Analysis (GSEA) [51].

### Cancer and healthy samples in a unified dataset

In the latest analyses, we processed data of samples unified from GTEx and The Cancer Genome Atlas (TCGA), this dataset was prepared and described in [52]. Normalized data were downloaded from [53].

We filtered samples in the analyses described in this manuscript, in particular, we sorted tissues by the number of samples available and got the eight tissues with the largest number of samples: Breast, Colon, Esophagus, Liver, Lung, Prostate, Stomach, and Uterus. We randomly selected 100 samples per tissue which we used to define the topic space, while all the other samples were used for training and validation. We set a cutoff above 100 000 FPKM (Fragments Per Kilobase Million) to reduce the number of edges and avoid overflows during the fitting procedure. This clipped the 0.2% of the links.

We collected the metadata through the GTEx portal and using the Genomic Data Commons tools [54] for the TCGA samples.

### Predictor on latent space

We built an NN predictor using topics as features, in other words, we trained a simpler model on a low-dimensional space instead of a complex model on the original ∼20 000 dimensional space.

The first step of this analysis was picking up 800 samples of 8 tissues from the unified dataset [53]. We fit the hSBM using this subset. In output, we obtained the topic distribution of samples.

At this point, we considered topics as features and *P*(topic|sample) as entries of the design matrix *X*. The entry *X*_*ij*_ is the *P*(topic_*j*_|sample_*i*_). The matrix needed further normalization to be fitted using Stochastic Gradient Descent later. The normalized matrix $\overline{X}$ was obtained by subtracting features means and dividing each feature by its range. The entries of this new matrix are $\overline{X_{ij}}=\frac{X_{ij}-\langle X_{ij\prime }\rangle _{j\prime }}{0.5*\left(\max _{j\prime } X_{ij\prime }-\min _{j\prime } X_{ij\prime }\right)}$.

The dataset was split into a training and a test set; the training set contains the 95% of the samples. This is quite unbalanced, but we were not going to use it to evaluate the performance of the model. Moreover, 25% of the training set was used as the validation set. The model consisted of a neural net with one hidden layer with 100 neurons activated by a *ReLU* function, the optimizer was set to Stochastic Gradient Descent. Finally, the output layer used a *softmax* activation to classify tissues.

We wanted to evaluate the performance on unseen samples, never retraining either topic modeling or the neural net. From the original dataset, we selected all the samples not involved in topic modeling. These were ∼5000 new samples never fitted by hSBM neither by the neural net. We projected this *unseen* samples into the topic space. To do this, we firstly selected the genes involved in topic modeling, the ones that passed the highly variable filter we imposed. The *P*(topic|sample) for the new samples were simply *P*(topic|sample) = Σ_gene_*P*(topic|gene)*P(gene|sample), in other words each expression array is multiplied to the matrix *genes*×*topics*. We now evaluate the NN performances in separating tissues on this new dataset and obtained an accuracy of 0.9273. The area under the curve (AUC) score is here 0.9852.

A similar experiment can be done, but considering ‘healthy’ and ‘diseased’ as labels. In this case, the output layer is simply a single neuron activated by a *sigmoid* function. The scores when predicting the status of the samples are 0.9474 for accuracy and 0.9762 for AUC.

The confusion matrix and the receiving operating characteristic (ROC) curves (reported in Fig. 11) were estimated using scikit learn [41]. The ROC curve represents the True Positive Rate or sensitivity ($\frac{TP}{TP+FN}$) versus the False Positive Rate or 1 − specificity ($\frac{FP}{FP+TN}$).

The whole predictor model was implemented using keras [55] and using TensorFlow as the backend.

We measured the K-Nearest Neighbor (K-NN) performance using the accuracy and the AUC scores. We applied K-NN setting n_neighbors to 5 and using *euclidean* metric.

## Results

### Discovering the structure of the transcriptome of human tissues with different topic modeling approaches

We start the analysis by applying different topic modeling techniques to a large-scale dataset of RNA-sequencing samples of healthy human tissues from the Genome Tissue Expression project (GTEx) [56]. The performance of topic models and commonly used clustering methods can be compared to the simple task of tissue separation. However, topic models have a probabilistic nature that provides an additional layer of information.

**Figure 1.**
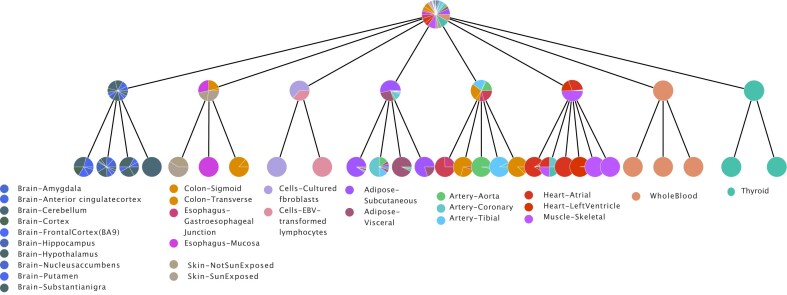
The hierarchy structure is represented as a tree. The first layer from the top is an all-samples node by construction. Then the algorithm found another node with all samples, at this point only the gene side of the network is partitioned. Then the algorithm found 8 clusters with a rough separation of tissues. From left to right the reader can identify a Brain cluster, a cluster with Colon, Esophagus, and Skin, a cluster with the label Cells (i.e. cultured fibroblasts and Epstein-Barr Virus-transformed lymphocytes), one mostly with Adipose tissue, one with Artery, one with Muscle and Hearth, one of Blood, and in the end one with Thyroid. In the next layers, the classification becomes finer, from Brain a Brain - Cerebellum cluster emerges, Skin and Colon are separated, Cells are split into Fibroblasts and Lymphocytes, Aorta, Coronary, and Tibial emerge, Hearth and Muscle are separated.

Figure 1 reports the results obtained using the topic modeling technique called hSBM [25]. The output of this model consists of blocks of samples (which we will denote as “clusters”) on one side, and blocks of genes (i.e. the topics) on the other side of a bipartite network. As a probabilistic method, these blocks can be fuzzy and eventually overlap. However, at this stage, we focus on not overlapping blocks to allow a straightforward comparison with clustering methods. Topic models also provide a probabilistic membership of samples to the topics. A mixture of topics can describe a sample via the probability distribution *P*(topic|sample) defined in Eq. 3 of the ‘Materials and methods’ section. Analogously, the gene membership to topics is probabilistic. The distribution *P*(gene|topic), defined in Eq. 4, captures the relative contribution of a gene to a specific topic. Moreover, the hSBM model has a hierarchical structure. The blocks can be organized in *L* layers, and blocks of layer *l* are nodes in layer *l* + 1 [36].

In principle, topic modeling algorithms should first recognize the basic tissue structure hidden in the expression profiles, and then recognize more fine-grained structures such as the sub-tissues. Figure 1 suggests that indeed this is roughly the case.

Besides hSBM, we ran the same analysis with other algorithms, specifically the well-known model LDA [11] and the more recent TM [26]. We also compared the cluster structure found by WGCNA [27, 43], which outputs *modules* of correlated genes, by using the standard implementation in its R package https://cran.r-project.org/package=WGCNA. The the ‘Materials and methods’ section reports the implementation details for each algorithm. Finally, we also tested hierarchical clustering on the same task [28]. We included it in the comparison since it is widely used thanks to its simplicity.

To reduce the complexity of the problem, we selected a subset of GTEx with 1000 samples from the 10 most represented tissues, and 3000 highly variable genes, as explained in more detail in the ‘Materials and methods’ section. In this setting, the output of hSBM has four layers with 1, 8, 29, 978 clusters at the sample level, and 22, 273, 2725, and 2880 topics, on the other side of the network. The first and last partitions are clearly not informative, while the two intermediate ones (8 and 29 clusters) are expected to capture the actual organization in tissues of the samples. In particular, the first one has a number of clusters close to the actual number of tissues in input, while the second partition (29 clusters) is expected to capture the subdivision of each tissue in more fine structures, for example distinguishing the cortex from the cerebellum in brain samples. Notice that the first partition of the genes into 22 topics happens when the samples are still all in the same cluster. This could be due to the asymmetry of the network. In fact, the number of genes is three times the number of samples. The second partition at the gene level, defining 273 topics, corresponds instead to the partition of samples in tissues.

hSBM is fully nonparametric: the hierarchical structure defined by the number of clusters and layers is automatically selected by the model [36]. Interestingly, the inferred hierarchical organization agrees with the hierarchical organization of tissues. For instance, from the cluster composed by *Brain* samples the following splitting leads to a sub-cluster composed by the *Brain - Cerebellum* samples. The possibility of separating brain samples from expression information was indeed previously observed by [56], when the dataset was released. Other examples of tissue and subtissue separations are reported in the caption of Fig. 1.

**Figure 2.**
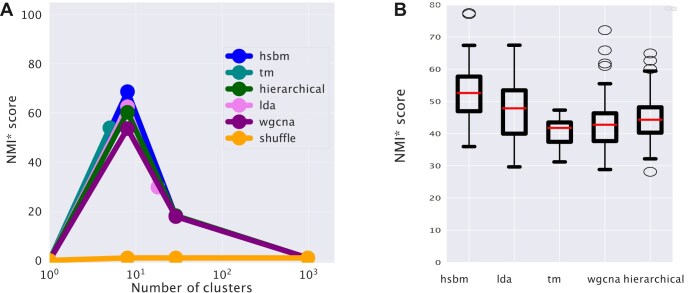
The *NMI* score of different algorithms. This score measures the agreement between the clusters obtained with the different algorithms and the ground truth sample partitioning defined by the tissues. **(A)** Looking at the maximum score reached at different levels of the hierarchy. All the algorithms reach a maximum score of around 10 clusters, which coincides with the number of tissues that we selected for the test. Topic modeling methods typically have the highest score. **(B)** The distributions of the normalized NMI scores corresponding to the resolution closer to the tissue structures for the different algorithms are reported as box plots. The variability is determined by the fluctuations in the NMI of random partitions of the same size that we use to define the normalized score NMI*.

We compare the performances of the different algorithms in Fig. 2 using the NMI as a measure of the clustering quality. This measure was proposed by [46] to evaluate topic modeling performances on synthetic corpora. Since the NMI is not null for a random partition (there may be some residual entropy), we chose to scale the NMI score to a simple random null model obtained by reshuffling labels and fixing the number and the size of clusters. We will refer to this normalized measure as NMI*. The ‘Materials and methods’ section reports all the details about the evaluation metrics.

Figure 2 shows that at the first hierarchical level, corresponding to tissue partitioning, all the algorithms show rather good performance values, with a NMI* in the 50–70 range. hSBM and LDA perform slightly better than the other algorithms, but the performance gain is not significant on this simple clustering task. TM seems unable to recover the hierarchical organization of the data, probably due to its more rigid structure, and stops at the first partition layer. While at the level of sample clustering, all the algorithms behave in a quite similar way, their substantial differences manifest in the definition of topics, as the next section explains.

### The effect of model assumptions and priors on the structure of the latent space

Topic modeling can also be interpreted as a projection of the data on a lower dimensional space in which the degrees of freedom are the topics. In this section, we investigate the structure of this latent low-dimensional space. In particular, we prove that different algorithms represent the data in latent spaces with very different properties, as a consequence of their different priors and inference procedures.

The structure of the topic space is defined by the topic distribution associated to samples. Specifically, the distribution *P*(topic|sample) for a sample can be considered as its coordinates in the low-dimensional topic space. We can then average over the samples belonging to a tissue to obtain *P*(topic|tissue) = ∑_sample ∈ tissue_*P*(topic|sample), i.e. the projection of a whole tissue in the topic space. Since a topic is nothing but an ordered list of genes, these probabilities define which genes play a relevant role in identifying the tissues and defining the structure of the topic space. Building a dataset composed by samples from different tissues, thus with a well known structure, should help the biological interpretation of topics and of the genes involved. Identifying samples from different tissues should not be challenging, given their distinct expression patterns. In fact, most algorithms perform comparably well on this task (Fig. 2). Consequently, one might expect that the latent topic structure inferred by various methods would be roughly similar.

**Figure 3.**
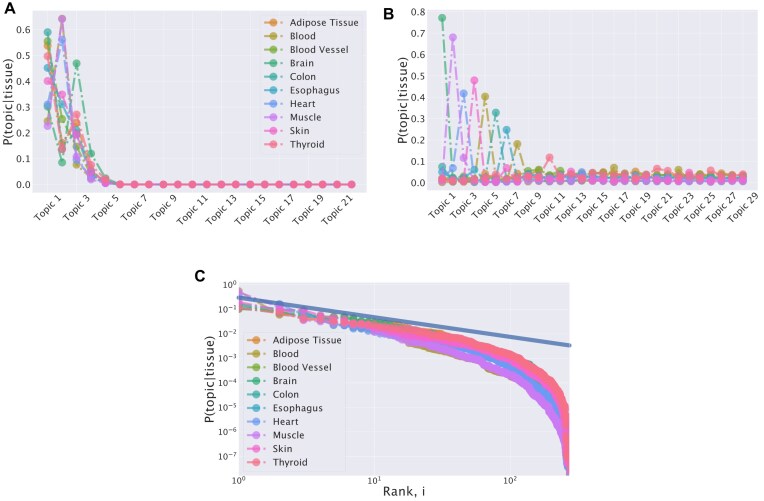
*P*(topic|tissue) for different tissues. The topic importance in samples of different tissues is reported in panel **(A)** for the hSBM and in panel **(B)** for LDA using a dataset composed of samples from 10 tissues of GTEx and selecting the highly variable genes. The trend of the reconstructed topic space is affected by the algorithm priors. In panel **(C)**, topics are ranked in every tissue so that the first point corresponds to the most expressed topic in that tissue. For convenience, the plot is reported on a log-log scale. The power-law structure, at least for low ranks, of the original data space is preserved in the hSBM topic space. The analogous plot for LDA would be trivially peaked due to the probability distribution induced by the Dirichlet prior.

Figure 3 reports *P*(topic|tissue) across tissues for two different algorithms. Figure 3A indicates that hSBM tends to find a common distribution for all tissues. Samples from all tissues are described by a mixture of a few important topics [i.e. with high *P*(topic|sample)], and several more specific but less present ones [with low *P*(topic|sample)]. The way the topics integrate on a global scale differentiates the tissues.

On the contrary, LDA leads to a radically different result due to its specific priors. The Dirichlet prior on which LDA is based forces the topic distribution towards more peaked solutions [11], as Fig. 3B clearly shows in our case. As a consequence, the identified topic are much more tissue specific, with no common highly represented topics. This result is particularly appealing in the biological context because it potentially offer an easy interpretation. A sample is often characterized by just one or a few topics, and thus by a relatively small set of associated genes. Nevertheless, this behavior is likely the result of the Dirichlet prior and, thus, the one-to-one association between topic and structure could not be actually present in the data. Similarly to LDA, TM, and WGCNA output a single layer of topics, with a structure of the topic space composed by a set of peaked distributions (see Supplementary Fig. S5).

Gene expression levels are typically power-law distributed in most datasets [22, 57], and indeed a power-law distribution also well describes the expression levels in all the tissues (Supplementary Fig. S6 in the Supplementary material). hSBM reflects this statistical property by reproducing an analogous distribution for the inferred topic structure (Fig. 3C). This model was indeed originally proposed to abandon the specific LDA priors in the linguistic context, where power-law word abundance distributions are also ubiquitous, and was then applied to RNA-sequencing data with similar motivations [16, 17]. Here, we directly show the effect of this change of prior on the inferred topic structure.

**Figure 4.**
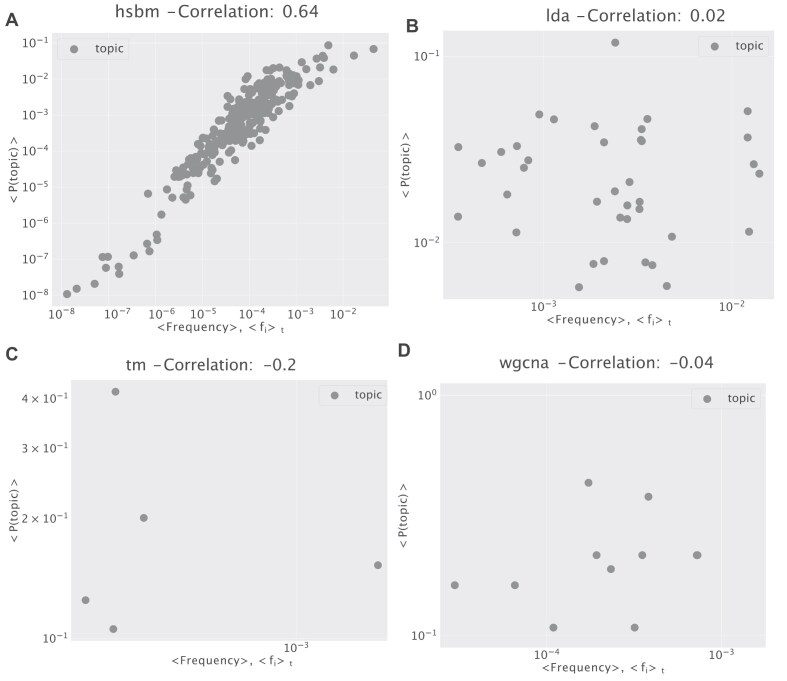
The average frequency of genes in each topic. We report the average normalized expression of genes in a topic: $\langle f_i\rangle _t = \frac{1}{|t|}\Sigma _{i \in t} \Sigma _{j=1}^{R} n_{ij} / R$, where *R* is the number of samples and *t* is a topic with |*t*| genes. We evaluate the correlation of this quantity with the frequency of topics in samples $f_t = \frac{1}{R} \Sigma _{s=1}^{R} P(\text{t} | \text{s})$. The different algorithms **(A)** hSBM, **(B)** LDA, **(C)** TM, and **(D)** WGCNA lead to very different correlation values. The topic distribution of hSBM is strongly correlated to the word distribution in the original data. This result resonates with a previous observation [25] about the widely different ‘disseminations’ of topics when using hSBM on texts. This result can be reproduced at any level of the output hierarchy. We report here the hierarchical level of each algorithm with a number of clusters close to the number of tissues in the dataset (10), and a sufficient number of points to estimate the correlations: hSBM has 273 topics and 8 clusters, LDA 38 topics, and 39 clusters. TM and WGCNA are not hierarchical models on the topic side, so we cannot select the number of topics.

The main difference in the structure of the inferred latent spaces can be observed using correlation analysis. We can measure the correlation between the typical expression of a gene in a topic 〈*f*_*i*_〉_*t*_ and the topic frequencies 〈*P*(topic)〉 in the samples. 〈*f*_*i*_〉_*t*_ is estimated by averaging the normalized expression of genes in each given topic, while 〈*P*(topic)〉 is the normalized abundance of topics in all samples. These two quantities are clearly correlated using hSBM, while this is not the case for LDA or other methods (Fig. 4). In other words, hSBM finds the tissue structure in the data by defining topics that typically contain genes with similar expression levels. This is not true for other methods. Again, this is a consequence of the different model priors, and in particular of the uniform prior implicitly present in hSBM. Which topic organization is more desirable can be debated, and it is probably context-dependent. However, it is important to keep in mind that the specific gene grouping is strongly influenced by the selected method, especially when attributing a biological interpretation to the topics.

In general, hSBM does no push towards few cluster-specific gene “markers”, but it rather organizes genes with similar expression values into topics and then uses the specific global combination of topics to cluster the samples. On the other hand, methods such as LDA tend to find topics composed by few genes with variable expression levels, and cluster of samples strongly associated to just one or few of these topics. While this is particularly appealing in terms of biological interpretation and subsequent experimental testing, it can be an artifact due to the model prior.

The impact of the different priors becomes even more evident when the analysis is restricted to housekeeping genes. These genes are, by definition, consistently expressed in every sample. Their global expression pattern can still have enough information to separate different tissues, but we do not expect to find housekeeping ‘markers’ of a tissue, i.e. housekeeping genes that are clearly differentially expressed in a specific tissue. Indeed, as expected, the distribution of hSBM topics is again power-law-like, and the obtained tissue separation is still quite accurate [the NMI* top scores (hSBM 43, LDA 47, TM 46, WGCNA 27, and hierarchical 35) are slightly smaller than the scores obtained with the standard gene selection presented in Fig. 1], but based on global differences in topic composition (Supplementary Fig. S7A). On the other hand, LDA identifies single over-represented topics in different tissues in this case as well, likely due to the influence of the Dirichlet prior (Supplementary Fig. S7B).

Biological processes result from complex interactions among potentially hundreds or thousands of genes. While reducing this complexity to a short list of ‘markers’ may be appealing, it can be overly simplistic and may overlook genes that, acting in synergy with those markers, play a causal role in the process. Such oversight can hinder the development of effective therapies by missing potential targets. [58].

### The relations between different tissues in the latent space

**Figure 5.**
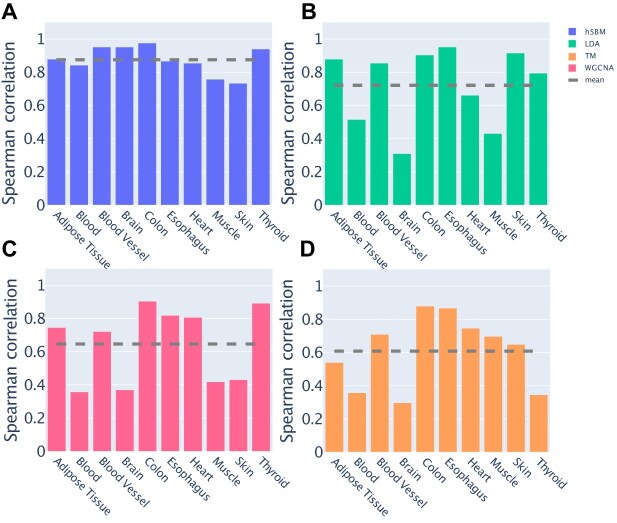
Spearman correlation between vectors of tissue distances calculated in the expression space or in the topic space. A comparison between different algorithms (**A**) hSBM, (**B**) LDA, (**C**) TM, (**D**) WGCNA is reported. The dashed lines correspond to the mean value across tissues. High correlation values indicate a conservation of the tissue relations in the topic space.

Since topic modeling can be viewed as a form of dimensionality reduction, a relevant question is how much of the structure from the original space is preserved in the low dimensional topic space. Our benchmark dataset is well suited to investigate this question. In fact, as reported originally by [56], there is a natural hierarchy of distances between tissues that can be observed at the level of gene expression patterns. We are interested in testing how much this hierarchy is preserved in the topic space created by different topic modeling techniques, in particular LDA and hSBM. Firstly, we define an ‘archetype’ per tissue by averaging the *P*(sample|topic) over all its available samples. We use these prototypical points to measure the distances between tissues in the topic space. Analogously, the same calculation can be performed in the original expression space. We can thus associate to each tissue two alternative vectors of distances from all the other tissues, one evaluated in the original data space, and one in the topic space. At this point, the Spearman correlation between these two vectors provides information about the conservation between the tissue relations. The correlation is 1 if the ranking of the distances is perfectly consistent after the projection in the topic space. As shown in Fig. 5A, the low dimensional projection of hSBM well conserves the tissue hierarchy in the expression space. Interestingly, this is only partially true for LDA (Fig. 5B), while with TM (Fig. 5C) and WGCNA (Fig. 5D) the tissue structure appears to be lost (this analysis cannot be done with hierarchical clustering since the original points are not projected in a low-dimension space).

### Different methods recover similar clusters but different topics

**Figure 6.**
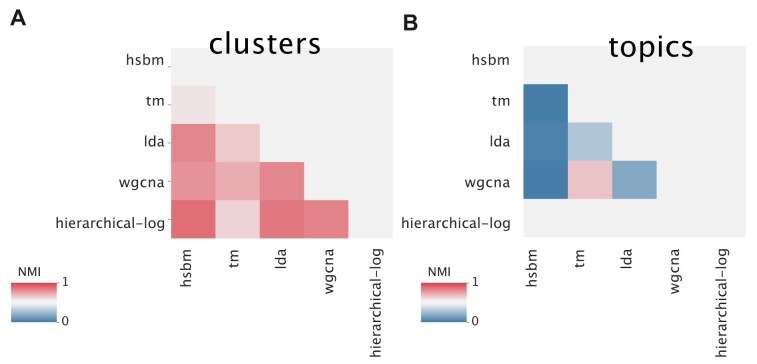
Similarity between the partitions obtained with different algorithms on the sample side **(A)** and on the gene side **(B)**. All the data are reported in Supplementary Tables S2 and S3 in the Supplementary materials. The similarity is evaluated using the NMI* score, as defined in the ‘Materials and methods’ section. Different algorithms build very similar sample partitions but tend to build nonoverlapping topics. Hierarchical clustering does not provide a topic structure, therefore it cannot be compared with other methods at the gene level. Most of the algorithms present a hierarchical structure, in this case, we compared them at a single point of resolution. We chose a resolution in which all algorithms present a compatible number of topics (hSBM 22, LDA 8, TM 5, WGCNA 21) and clusters (hSBM 29, LDA 28, TM 5, WGCNA 30, hierarchical 29).

The phenotypic differences observed across tissues are strongly reflected in their gene expression profiles. In fact, nearly every algorithm tested was able to effectively distinguish between samples from different tissues. This is confirmed by the high NMI [45] (see the ‘Materials and methods’ section for a detailed discussion of the model evaluation measures) values obtained, using the known tissue structure as ground truth (Fig. 2). Indeed, different algorithms converge to very similar partitions at the level of samples, at least at the coarse resolution of tissues (Fig. 6A). However, as discussed in the previous sections, the topic spaces generated by alternative models exhibit structural differences. We now directly assess the overlap in the genes that the various models prioritize to achieve tissue separation.

Figure 6B reports the agreement between the partitions of genes obtained with different models. The low NMI scores show that the overlap between the gene clusters is quite low. Therefore, the different model priors not only lead to distinct structures of the topic space but also produce varying associations between genes and topics. This implies that the gene sets linked to specific tissues are highly model-dependent.

In general, machine learning methods, such as topic modeling, leverage statistical associations to perform a task without any domain knowledge. These statistical associations obviously do not necessarily correspond to causal relations [59–61]. Given that the number of genes often exceeds the number of samples and that gene expression levels are frequently correlated [e.g. genes regulated by the same transcription factor (TF)], multiple combinations of genes could effectively correlate with the tissue structure. Consequently, it is not surprising that different models, each with distinct priors, may focus on different gene sets for clustering the samples. However, the substantial differences in the gene sets associated with the samples serve as a caution to users: these associations should not be overinterpreted, and the biases inherent in each model should be carefully evaluated.

### GSEA confirms the biological relevance of the topics

**Figure 7.**
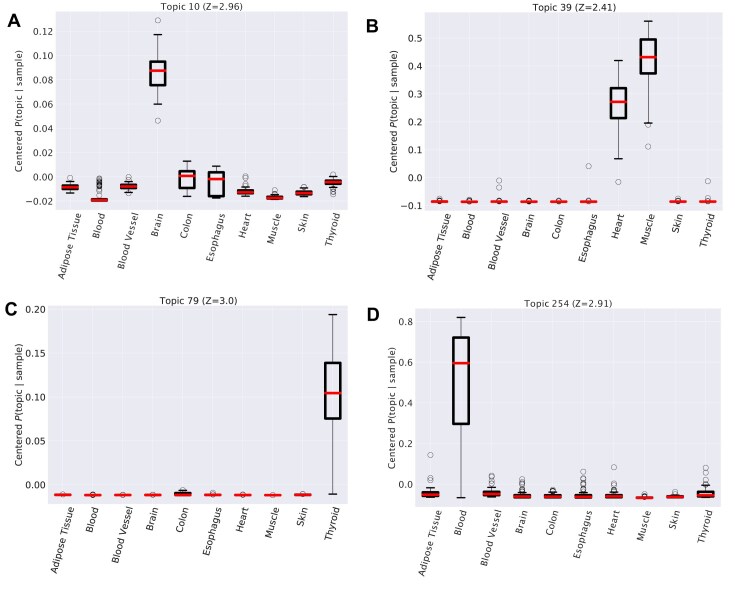
Distribution of centered $\bar{P}(\text{topic} | \text{sample})$. We grouped $\bar{P}(\text{topic} | \text{sample})$ for all samples belonging to the same tissue in each box. Each subpanel refers to a specific topic. This value reveals, in the probability distributions, trends correlated with the tissues. We reported in this panel some of the more informative topics. In Table 1 we reported gene ontologies of genes in these topics.

Topics are essentially lists of genes and thus GSEAs can be used to identify the biological features associated to topics. We start this analysis from the tissue structure identified by hSBM. We first build a centered version $\overline{P}(\text{topic} | \text{tissue})$ of the probability distribution *P*(topic|tissue) by subtracting the mean probability value. This centered version highlights the topic that are over-represented in a given tissue as displayed in Fig. 7.

We can now isolate the topics whose centered probability is higher in one or more tissues using a z-score to evaluate the significance of these deviations. Finally, we can apply a GSEA [51] to the gene lists associated the significant topics. An analogous procedure was previously used to interpret the biological meaning of topics in the context of cancer transcriptomics [16]. Illustrative examples of this analysis are reported in Fig. 7 and in Table 1. In many cases, a single topic is over-represented in a tissue and thus should be representative of the tissue biological properties. This is the case of topic 10 in Fig. 7A, which is strongly associated to brain tissues. The genes in the topic are indeed enriched in neuronal Gene Ontology (GO) categories (see the first entry of Table 1). In some other cases, a single topic is enriched in more than one tissue, probably due to common biological pathways or functions in the two tissues. This is the case of topic 39, which is associated both to Muscle and Hearth (Fig. 7B), and accordingly to the keywords “myosine”, “myogenesis”, and “muscle contraction”. Other examples are reported in Fig. 7 with the corresponding enrichment results in Table 1.

**Table 1.** Gene ontology enrichment tests performed on the gene lists (number of genes reported in brackets) associated to different topics using GSEA [51]

Term	False Discovery Rate (FDR) q-value
Topic 10 (34)	
Neuronal cell body	8.07*e* − 5
Axonogenesis	2.25*e* − 4
Topic 39 (18)	
HALLMARK_MYOGENESIS	6.81*e* − 28
Muscle contraction	3.15*e* − 24
Topic 46 (33)	
GO_EXTRACELLULAR_MATRIX	1.95*E* − 10
NABA_MATRISOME	3.35*E* − 10
Topic 79 (10)	
GO_THYROID_HORMONE_METABOLIC_PROCESS	7.94*E* − 4
GO_ENDOCRINE_SYSTEM_DEVELOPMENT	4.9*E* − 2
Topic 254 (5)	
GO_HAPTOGLOBIN_BINDING	2*E* − 6
GO_HAPTOGLOBIN_HEMOGLOBIN_COMPLEX	2*E* − 6

Interestingly, some topics are common to several (or all) tissues. In the context of natural language processing, these topics have been characterized as ‘common topics’ containing less informative words, such as articles [25], which are present in all texts. Analogously, in our context, these common topics are enriched in general GO terms, which are shared by all the tissues. This is the case, for instance, of topic 46 (see the third line of Table 1 which is enriched in keywords like ‘extracellular matrix’ which can be associated to different tissues.

We report the above examples of functional annotations, although somewhat generic, primarily as a sanity check for our analysis. However, the hSBM topic organization can also reveal new biological insights, particularly regarding the regulatory pathways driving tissue separation. Notably, an examination of the gene content in certain topics shows enrichment for targets of specific TFs. In the Supplementary Material, we provide a detailed example highlighting the role of three key TFs—MEF2, AP4, and SRF—in the organization of Blood and Muscle cells [62–64]. At the higher hierarchical level (Topic 6 of level 3), all three TFs are strongly enriched, with a clear association of the topic to Blood and Muscle samples. At the subsequent hierarchical level, the genes in Topic 6 split into several sub-topics, each characterized by distinct target enrichment and tissue associations. This differentiation of regulatory patterns is supported by existing literature. Consistent with the general behavior of hSBM, this differentiation is not a rigid separation but a gradual shift in enrichment content: several genes are shared targets of different TFs contributing to tissue formation, likely with distinct functional roles. A more detailed discussion of this example is provided in the Supplementary Material and illustrated in Supplementary Fig. S8.

We performed the same functional analysis for the topics identified by the other three algorithms. All the results are presented in Supplementary Figs S9 (LDA), Supplementary Fig. S10 (TM), and Supplementary Fig. S11 (WGCNA) of the Supplementary material. The GO enrichment is relatively strong for WCGNA and TM topics, while slightly weaker for LDA ones. LDA does not naturally define the topics as gene lists, instead its output is a probability distribution whose support is the whole gene repertoire. As discussed in the ‘Materials and methods’ section, we select the 20 genes with the higher associated probability given a topic (20 the typical number of genes per topic found by hSBM).

An issue related to the WCGNA results is that the gene list associated with a topic is in general very large (one order of magnitude larger than the average hSBM list) making the topics much less specific. On the contrary, TM outputs on this dataset a small (i.e. 5) number of topics to identify a one-to-one relation between topics and tissues. Possibly, these problems could be made less severe with a suitable choice of hyperparameters. However, as we mentioned in the introduction, in order to make a fair comparison with parameter-free methods such as hSBM and to avoid overfitting we did not perform model-specific explorations of hyperparameter values, and adopted the standard settings.

Interestingly, even if the topics found by different methods are very different, as shown in the previous sections, the associated gene lists often shows significant enrichment for gene ontologies that are compatible with the tissue structure (see Supplementary Figs S9–S11).

### The use of topic modeling to distinguish healthy from cancerous tissues

As an additional relevant benchmark dataset, we combined samples from healthy tissues from GTEx [56] and cancerous tissues from TCGA [52]. As a consequence, two main strucures are present in this dataset: the tissue structure and the distinction between healthy and cancer tissues.

**Figure 8.**
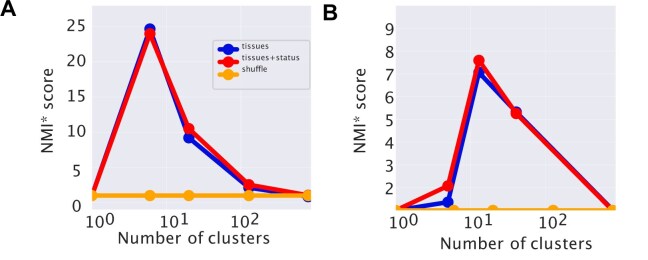
Identifying healthy and cancer tissues. We report the NMI* scores (see the ‘Materials and methods’ section) for hSBM **(A)** and for LDA **(B)** when applied to a dataset combining healthy and cancerous samples from 20 different tissues. The score is evaluated with respect to the tissue structure (blue lines, lower at high number of clusters) or concerning the tissue structure with the additional status annotation (red lines, higher at high number of cluster).

The results of applying hSBM and LDA to this dataset are reported in Fig 8A and B, respectively. We display the NMI* levels evaluated with respect to the tissue structure only (blue lines), or to the tissue structure with the additional partition due to status, i.e. healthy versus cancerous (red lines). The task of retrieving even just the tissue structure is now more challenging due to the additional variability introduced by the presence of cancerous samples. Indeed, the NMI* values are lower than those reported in Fig. 2. In this more complex setting, hSBM outperforms LDA in discovering both structures, further confirming its potential in the context of cancer transcriptomics [16].

The lower resolution level is dominated by the tissue organization. In fact, hSBM typically finds a number of clusters close to the number of tissues (10) and the NMI is higher if evaluated with respect to the tissue structure. At the subsequent layer, the algorithm further separates reasonably well the healthy tissues from the cancerous ones. Indeed, the red line (referring to the partitioning with health status) becomes higher than the blue line, where the number of clusters is generally close to 20 as expected (Fig 8A). This effect is less evident using LDA (Fig 8B).

### Robustness to data pre-processing and gene selection

Data pre-processing and gene selection procedures to reduce the number of features are standard practices in the analysis of RNA-sequencing data [65]. However, most protocols do not have a clear theoretical motivation and can potentially affect downstream analysis and conclusions. In this section, we focus on the often-used data logarithmic transformation and on gene selection procedures to analyze their effect on the results of topic modeling algorithms.

#### Topic modeling algorithms do not need data log-transformation

**Figure 9.**
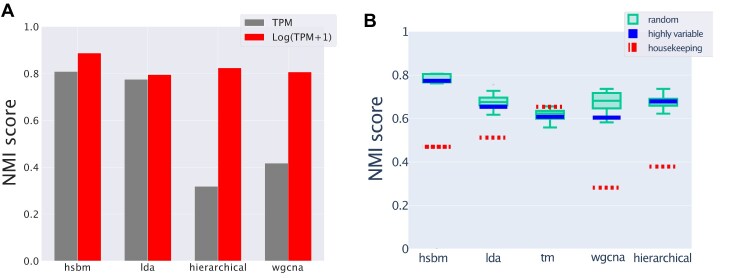
Robustness to data pre-processing. (**A**) The performances in terms of NMI of different algorithms in retrieving the tissue structure when operating on *TPM* expression values (gray) or on log-transformed data using log(*TPM* + 1) (red, bars on the right). (**B**) Comparing different gene selections. We reported the NMI score of the hierarchy level with the number of clusters more similar to 10 (the real and expected one) (for some algorithms, e.g. LDA, it is not possible to force the number of clusters, that is why it may vary. See the ‘Materials and method’ section for details). The red dashed lines represent the particular choice of 3000 housekeeping genes from ref. [49]. The scores obtained using the standard selection of highly variable genes are reported as blue lines. The green boxes report the scores for different algorithms when choosing a random subset of genes as features (for each selection and each algorithm we chose the score where the algorithm found a number of clusters more similar to 10). We use directly the NMI, without the normalization with respect to a random partition, since the number of clusters is quite constant in this analysis.

The mRNA counts from RNA-sequencing experiments are the result of a sampling process, thus their variability is due to a combination of biological and technical sources [22, 66]. For this reason, the data are typically first normalized to reduce the sampling effect. Moreover, the data are often log-transformed [67] with the underlying hypothesis that fold-change variations are more relevant than absolute changes. In fact, the euclidean distance of sample *j* from sample *j*′ is defined as $\sqrt{\sum _i\left(n_{ij}-n_{ij\prime }\right)^2}$, but it becomes $\sqrt{\sum _i\left(\log _2\left(\frac{n_{ij}}{n_{ij\prime }}\right)\right)^2}$ with the log-transformation, making the relative fold-change $\frac{n_{ij}}{n_{ij\prime }}$ the only relevant quantity. By affecting how the distances are evaluated, this pre-processing procedure can affect the results of clustering methods. Therefore, we want to compare the performances of different algorithms using samples in the original data space (using the standard TPM values) or samples in the log-transformed space. Figure 9 summarizes this comparison. It is clear that topic modeling methods, both hSBM and LDA, do not need this pre-processing step to obtain significant scores in the task of finding the tissue structure in the dataset. On the other hand, there is a relevant drop in performance for both WCGNA and hierarchical clustering if the log-transformation is not applied. TM was not included in this analysis because it considers a network of genes based on co-occurences that are unaffected by a log-transformation. A gene is present in a sample if TPM > 0 and this is equal to log_2_(TPM + 1) > 0.

The log-transformation has several potential pitfalls. For example, the zero count values have to be replaced by a small constant before the transformation, and this can introduce artifacts in sparse datasets. Moreover, the log-transformation compresses differences at higher expression levels that could be biologically relevant. Therefore, it is important to know which algorithms can actually operate reliably in both expression spaces and our analysis shows that this is the case for topic modeling methods.

#### Robustness to gene selection

Another standard procedure in the analysis of RNA-sequencing data is gene selection [48]. Reducing the number of features is often necessary to speed up downstream analysis, and can also potentially eliminate less informative features that can act as confounding factors. This section discusses the robustness of different algorithms with respect to feature selection by considering the two extreme cases of random gene selection and of selection of housekeeping genes. While typically an arbitrary number of highly variable genes is used (and this is the selection criterium we adopted so far), these more radical criteria can better highlight the algorithms’ dependences on gene selection.

Actually, the ability of different algorithms in retrieving the tissue structure when using highly-variable genes or a random selection of genes is not significantly different (Fig. 9B). This result suggests that the information about the different tissue phenotypes is redundantly present in the expression profiles. Therefore, many different subsets of features can be used without significant differences.

Housekeeping genes are instead expressed in nearly every sample and typically exhibit high expression levels and low variability. Therefore, by selecting only housekeeping genes we are defining the more difficult task of retrieving the tissue structure using small and collective differences in otherwise quite conserved features. Figure 9B shows that topic modeling algorithms, can still reasonably well reconstruct the tissue structure in this setting (NMI scores as red dashed lines), while standard clustering algorithms, such as WGCNA or hierarchical clustering, have a more evident performance drop.

### Uncovering structures in single-cell RNA-sequencing data

**Figure 10.**
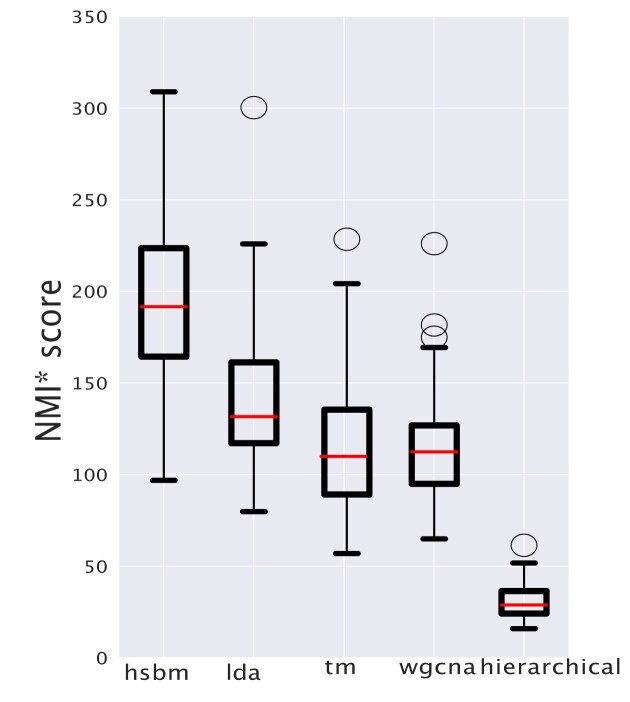
NMI* score relative to the clustering of single-cell data. We estimated the NMI scaled by the value corresponding to random partitions for all the algorithms considering their ability to separate cells from different organs. The general ranking with hSBM and LDA slightly outperforming other methods, is compatible with the results obtained using bulk data (Fig. 1).

We have focused so far on bulk RNA-sequencing data, but recently single-cell sequencing techniques are revolutionizing the field and providing a plethora of large-scale datasets [1] and topic modeling techniques can be also applied in this context [15, 18]. The main difference in the statistical properties of single-cell RNA-seq data with respect to bulk data is the data sparsity and the higher level of noise, due to the technical variability induced by the low levels of RNA in single cells and by the inherent stochasticity of gene expression, which is not averaged out as in bulk datasets. This section compares the different algorithms in the task of uncovering the organ structure in a sparse single-cell RNA sequencing dataset. In particular, we constructed a dataset that includes cells from four main organs from the Mouse Cell Atlas [68] and belonging to the five most represented cell types in each organ. Figure 10 reports the distributions of normalized NMI* scores for all the algorithms focusing on their ability to separate organs (a more comprehensive analysis is reported in Supplementary fig. S3). Also in the case of single-cell data, topic models can identify the hidden macro structure and hSBM and LDA seems to slightly outperform other methods. Again different methods converge on a similar clustering by using a different latent variable structure. In fact, Supplementary fig. S12 reports the analogous of Fig. 3 for the single cell scenario. The results are consistent with the bulk case, with hSBM retrieving global patterns of topic compositions, while LDA tends to associate a single topic to each single organ.

### Sample classification in the latent space

Topic models can also be interpreted as dimensionality reduction techniques. In fact, they define an embedding space (i.e. the topics) in which the data can be projected. A simple NN can be trained in a supervised manner to perform, for example, healthy versus tumor classification of samples represented in this embedding space. The idea is to use the unsupervised learning of a topic model to learn suitable sample representations, and then build a classifier in this representation space to annotate new samples. This procedure is analogous to the unsupervised pre-training that has been adopted in several deep learning projects [69].

In practice, each sample is only defined by a vector of its topics, reducing the ≃20 000 gene features to ≃1800 topics. The number of topics depends on the selected hierarchical level of hSBM and sets the dimension of the input space for the NN. We can thus use a simpler and faster-to-train model in the topic space without losing explainability since, as shown in the previous sections, topics can be associated to biological functions with GSEA.

**Figure 11.**
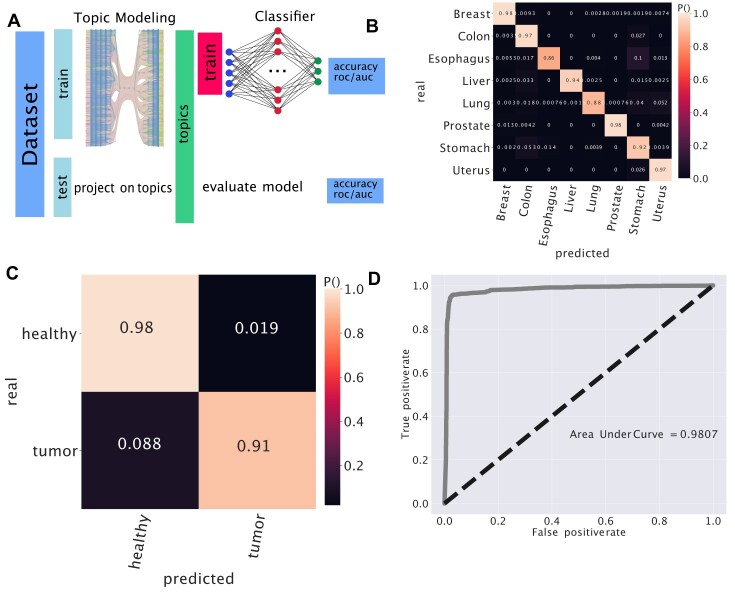
Prediction on the topic space. (**A**) Topology of the experiment. (**B**) The confusion matrix of the experiment involving tissues. (**C**) We run a similar experiment but try to predict if a tissue is healthy or diseased. The setting was the same as the tissue except for the tuning of some hyper-parameters and the fact that the last layer uses sigmoid as an activation function. In the figure the confusion matrix. (**D**) The panel shows the ROC curve. We considered *positive* healthy samples.

Using the topic space defined by hSBM, we trained an NN for two classification tasks: tissue separation and healthy versus cancerous sample. A standard cross-validation setup has been adopted, as presented in the cartoon of Fig. 11A. We also compared our results with a simpler K-NN classifier that can assign the labels using the sample position in the original expression space.

The first task is to classify the tissue origin of new samples (not previously used to define the topic space) by first projecting them in the topic embedding space. A small NN trained in this space obtains an accuracy of 0.93 on this task. Figure 11B reports the confusion matrix for this setting. Since the task is not particularly complex, a K-NN can be used to assign tissue labels with an accuracy around 0.8 in the original data space, and around 0.9 in the log-transformed expression space. Similar performance values can be obtained by training the NN in the original expression space (the full performance metrics are reported in Supplementary Tables S4 and S5). However, the advantage of the projection in the low-dimensional topic space is that we can train much smaller models with similar performance values and interpretable results. Moreover, as the size of expression datasets increases, the information extracted by the unsupervised pre-training with topic modeling is expected to continuously increase the performance on complex downstream tasks, even if defined by very small annotated datasets for supervised training.

The second task we tested was the ability to classify healthy versus cancerous samples. An NN operating in the topic embedding space obtains an accuracy of 0.95 and an AUC score of 0.98. The confusion matrix and the ROC associated to this binary classification problem are reported in Fig. 11C and D.

This analysis is a proof of concept that a simple NN can be used successfully in the structurally rich but low dimensional topic space for different downstream classification tasks.

## Conclusions

Topic modeling is a simple yet powerful unsupervised learning technique that can be naturally applied to transcriptomic data, leveraging on the strong statistical analogies with datasets of natural language [16, 22]. While few applications in this context have been explored [3, 13, 16, 17], we are still at the early stages of understanding its full capabilities and possible pitfalls. By comparing different topic models and standard clustering algorithms on a benchmark dataset with a partially known internal structure, we demonstrated that topic modeling can cluster expression samples as effectively as traditional methods. However, topic models offer a richer probabilistic framework, allowing for the extraction of latent variables and their associations with genes in a probabilistic manner. This framework also provides a graded sample membership rather than a mere grouping. Moreover, topic models appear to be generally robust with respect to data preprocessing and feature selection and can be used as dimensionality reduction methods.

Each topic model employs different priors. We have shown how these priors strongly influence the topics identified. This is especially relevant for transcriptomic data, in which relatively few samples are embedded in a very high-dimensional expression space. As a consequence, there are potentially many unconstrained degrees of freedom, which makes the choice of statistical priors particularly important. The presented results underscore the need to consider these priors carefully when selecting a topic modeling approach, as they shape the model focus and its ability to uncover biologically relevant structures.

Among the tested topic models, the hSBM offers several distinct advantages. It does not require adjustable parameters by automatically detecting the number of topics and naturally provides a hierarchical structure of the output. Moreover, our results demonstrate that hSBM captures the ubiquitous power-law distribution of gene expression values, projecting it onto an analogous topic distribution. Additionally, hSBM maintains the hierarchical relationships between hidden structures (the tissue structure in our case) in the embedding space. These features make it particularly well suited for capturing potentially complex structural relations in transcriptomic data.

Despite its advantages, hSBM has some limitations. In particular, it relies on time-consuming Monte Carlo simulations, which makes it slower compared to models like LDA. LDA, with its Dirichlet prior, may be particularly suitable for identifying short lists of ‘marker’ genes due to its tendency to identify single, over-represented topics within sample clusters. In contrast, hSBM naturally emphasizes global rearrangements of expression patterns, likely due to its ‘flat’ prior. Therefore, using hSBM and LDA in tandem could offer complementary insights, with LDA supporting marker extraction and hSBM providing a broader view of the overall structure.

We can summarize the main results of this paper into a few key guidelines:

- If the primary goal is to cluster samples or individual cells, classic clustering algorithms are computationally efficient, easier to interpret, and generally produce similar partitions to topic models, at least for datasets with naturally well-defined clusters, such as those studied here.
  However, if the objective is fuzzy clustering with probabilistic membership, topic models offer a good alternative. Additionally, topic models are generally preferable when associating gene sets with clusters. Indeed, these models naturally define a probabilistic relation between topics and clusters, and topics are essentially collections of genes.
- When there is a need to identify a small set of genes or markers associated with a cluster of samples or cells, the LDA prior naturally encourages this outcome. This is particularly useful when selecting a small set of marker genes for diagnostic purposes or experimental testing. However, our detailed analysis suggests that it is important to recognize that such associations are often influenced by intrinsic algorithmic biases and may not necessarily indicate a causal relationship.
- If the goal is to understand the complex interplay between genes underlying the partitioning of samples, hSBM is naturally suited for this purpose. Unlike topic models such as LDA, hSBM does not impose a specific bias toward sparse topic representation in clusters or sparse gene representation in topics. However, the main drawback of hSBM compared to LDA is its higher computational cost due to the Monte Carlo procedure.
- Note that the ‘flat’ prior implicitly present in hSBM leads to topics in which genes tend to have similar expression levels. As a result, the algorithm typically identifies a few topics consisting of highly expressed genes, which correspond to articles in text analysis and are often enriched in housekeeping genes in transcriptomics. The remaining topics are primarily composed of lowly expressed genes. Since hSBM considers global expression patterns,it is more robust than faster methods with respect to data normalization and gene selection procedures.
- We have shown that clusters are preserved across a range of quite different algorithms. Applying a consensus algorithm can, in principle, help remove even subtle noise from the clustering outcome.
- On the other hand, due to differences in model assumptions and the common disproportion between the number of samples and the number of genes, different algorithms identify different sets of genes to explain even very similar sample partitionings. Therefore, if multiple methods are applied to the same problem, their convergence on a similar set of genes suggests the presence of a strong association signal. However, they often converge on poorly overlapping sets of genes, which may still be associated with similar biological processes or pathways due to redundancy and correlations present in expression profiles.
  By considering the union of all genes associated with a given cluster across different algorithms, one can construct a comprehensive pool of genes for further investigation.

Topic modeling is still an active research area. In particular, there are recent developments of mathematical descriptions based on the stochastic block model that are still waiting to be adapted and tested for genomic data. For instance, merge-split Markov chains [70] or Bayesian nonparametric formulations [71] could lead to improvements in the performance of hSBM-like algorithms. Moreover, in this work, we ran hSBM with multiple initializations and chose the one with the shortest description length. However, the recently proposed technique based on consensus [72] could be an interesting alternative to explore.

## Supplementary Material

lqaf049_Supplemental_File

## Data Availability

Code and notebooks to reproduce our analyses are available from https://zenodo.org/records/15187437 [73], Topic and Cluster names (i.e. Cluster 1, Topic 1) may vary between the draft and the repository. We changed them for writing purposes.

## References

[1] Shapiro E, Biezuner T, Linnarsson S Single-cell sequencing-based technologies will revolutionize whole-organism science. Nat Rev Genet. 2013; 14:618–30. 10.1038/nrg3542. 23897237

[2] Han X, Zhou Z, Fei L et al. Construction of a human cell landscape at single-cell level. Nature. 2020; 581:303–9. 10.1038/s41586-020-2157-4. 32214235

[3] Dey KK, Hsiao CJ, Stephens M Visualizing the structure of RNA-seq expression data using grade of membership models. PLoS Genet. 2017; 13:e1006759 10.1371/journal.pgen.1006599. 28333934 PMC5363805

[4] Morelli L, Giansanti V, Cittaro D Nested Stochastic Block Models applied to the analysis of single cell data. BMC Bioinformatics. 2021; 22:576 10.1186/s12859-021-04489-7. 34847879 PMC8630903

[5] Ashley EA Towards precision medicine. Nat Rev Genet. 2016; 17:507–22. 10.1038/nrg.2016.86. 27528417

[6] Kolodziejczyk A, Kim JK, Svensson V et al. The technology and biology of single-cell RNA sequencing. Mol Cell. 2015; 58:610–20. 10.1016/j.molcel.2015.04.005. 26000846

[7] Kotliar D, Veres A, Nagy MA et al. Identifying gene expression programs of cell-type identity and cellular activity with single-cell RNA-Seq. eLife. 2019; 8:e43803 10.7554/eLife.43803. 31282856 PMC6639075

[8] Kiselev VY, Andrews TS, Hemberg M Challenges in unsupervised clustering of single-cell RNA-seq data. Nat Rev Genet. 2019; 20:273–82. 30617341 10.1038/s41576-018-0088-9

[9] Kohane IS The twin questions of personalized medicine: who are you and whom do you most resemble?. Genome Med. 2009; 1:4 10.1186/gm4. 19348691 PMC2651581

[10] Biondo M, Cirone N, Valle F et al. The intrinsic dimension of gene expression during cell differentiation. bioRxiv5 December 2024, preprint: not peer reviewed 10.1101/2024.08.02.606382. PMC12401001 40889499

[11] Blei DM, Ng AY, Jordan MI Latent Dirichlet Allocation. J Mach Learn Res. 2003; 3:993–1022. 10.5555/944919.944937.

[12] Zhang Y, Khalilitousi MS, Park YP Unraveling dynamically encoded latent transcriptomic patterns in pancreatic cancer cells by topic modeling. Cell Genom. 2023; 3:100388 10.1016/j.xgen.2023.100388. 37719139 PMC10504675

[13] Yang Q, Xu Z, Zhou W et al. An interpretable single-cell RNA sequencing data clustering method based on Latent Dirichlet Allocation. Brief Bioinform. 2023; 24:bbad199 10.1093/bib/bbad199. 37225419 PMC10359080

[14] Sun D, Liu Z, Li T et al. STRIDE: accurately decomposing and integrating spatial transcriptomics using single-cell RNA sequencing. Nucleic Acids Res. 2022; 50:e42 10.1093/nar/gkac150. 35253896 PMC9023289

[15] Segura MLRT, Moussa EA, Garabello E et al. A 3D transcriptomics atlas of the mouse nose sheds light on the anatomical logic of smell. Cell Rep. 2022; 38:110547 10.1016/j.celrep.2022.110547. 35320714 PMC8995392

[16] Valle F, Osella M, Caselle M A topic modeling analysis of TCGA breast and lung cancer transcriptomic data. Cancers. 2020; 12:3799 10.3390/cancers12123799. 33339347 PMC7766023

[17] Valle F, Osella M, Caselle M Multiomics topic modeling for breast cancer classification. Cancers. 2022; 14:1150 10.3390/cancers14051150. 35267458 PMC8909787

[18] Malagoli G, Valle F, Barillot E et al. Identification of interpretable clusters and associated signatures in breast cancer single-cell data: a topic modeling approach. Cancers. 2024; 16:1350 10.3390/cancers16071350. 38611028 PMC11011054

[19] Kazwini NE, Sanguinetti G SHARE-Topic: Bayesian interpretable modeling of single-cell multi-omic data. Genome Biol. 2024; 25:55 10.1186/s13059-024-03180-3. 38395871 PMC10885556

[20] Pizzini L, Valle F, Osella M et al. Topic modeling analysis of the Allen Human Brain Atlas. Sci Rep. 2025; 15:6928 10.1038/s41598-025-91079-9. 40011617 PMC11865453

[21] Mazzolini A, Gherardi M, Caselle M et al. Statistics of shared components in complex component systems. Phys Rev X. 2018; 8:21023 10.1103/PhysRevX.8.021023.

[22] Lazzardi S, Valle F, Mazzolini A et al. Emergent statistical laws in single-cell transcriptomic data. Phys Rev E. 2023; 107:44403 10.1103/PhysRevE.107.044403. 37198814

[23] Mazzolini A, Caselle M, Osella M Ranking nodes in bipartite systems with a non-linear iterative map. Commun Phys. 2025; 8:148 10.1038/s42005-025-02073-6.

[24] Fortunato S, Hric D Community detection in networks: a user guide. Phys Rep. 2016; 659:1–44. 10.1016/j.physrep.2016.09.002.

[25] Gerlach M, Peixoto TP, Altmann EG A network approach to topic models. Sci Adv. 2018; 4:eaaq1360 10.1126/sciadv.aaq1360. 30035215 PMC6051742

[26] Lancichinetti A, Sirer MI, Wang JX et al. High-reproducibility and high-accuracy method for automated topic classification. PhysRev X. 2015; 5:011007 10.1103/PhysRevX.5.011007.

[27] Langfelder P, Horvath S WGCNA: an R package for weighted correlation network analysis. BMC Bioinformatics. 2008; 9:559 10.1186/1471-2105-9-559. 19114008 PMC2631488

[28] Jr JHW Hierarchical grouping to optimize an objective function. J Am Stat Assoc. 1963; 58:236–44. 10.1080/01621459.1963.10500845.

[29] Schumacher C, Vose MD, Whitley LD The no free lunch and problem description length. Proceedings of the Genetic and Evolutionary Computation Conference (GECCO-2001). 2001; San Francisco CA, USA Morgan Kaufmann 565–70.

[30] Rich JM, Moses L, Einarsson PH et al. The impact of package selection and versioning on single-cell RNA-seq analysis. bioRxiv11 April 2024, preprint: not peer reviewed 10.1101/2024.04.04.588111. 41903535

[31] Ding J, Condon A, Shah SP Interpretable dimensionality reduction of single cell transcriptome data with deep generative models. Nat Commun. 2018; 9:2002 10.1038/s41467-018-04368-5. 29784946 PMC5962608

[32] Grønbech CH, Vording MF, Timshel PN et al. scVAE: variational auto-encoders for single-cell gene expression data. Bioinformatics. 2020; 36:4415–22. 10.1093/bioinformatics/btaa293. 32415966

[33] Lonsdale J, Thomas J, Salvatore M et al. The genotype-tissue expression (GTEx) project. Nat Genet. 2013; 45:580–5. 10.1038/ng.2653. 23715323 PMC4010069

[34] Guo G MCA DGE data. Figshare. 2018; 8:5435866 10.6084/m9.figshare.5435866.v8.

[35] Peixoto TP The graph-tool python library. Figshare. 2014; 1:1164194 10.6084/m9.figshare.1164194.

[36] Peixoto TP Hierarchical block structures and high-resolution model selection in large networks. Phys Rev X. 2014; 4:011047 10.1103/PhysRevX.4.011047.

[37] Peixoto TP Efficient Monte Carlo and greedy heuristic for the inference of stochastic block models. Phys Rev E. 2014; 89:12804. 10.1103/PhysRevE.89.012804 24580278

[38] Peixoto TP Model selection and hypothesis testing for large-scale network models with overlapping groups. Phys Rev X. 2015; 5:11033 10.1103/PhysRevX.5.011033.

[39] Peixoto TP Nonparametric Bayesian inference of the microcanonical stochastic block model. Phys Rev E. 2017; 95:12317. 10.1103/PhysRevE.95.012317 28208453

[40] Peixoto TP Doreian P, Batagelj V, Ferligoj A Bayesian stochastic blockmodeling. Advances in Network Clustering and Blockmodeling. 2019; USA John Wiley & Sons Ltd 289–332. 10.1002/9781119483298.ch11.

[41] Pedregosa F, Varoquaux G, Gramfort A et al. Scikit-learn: machine learning in Python. J Mach Learn Res. 2011; 12:2825–30.

[42] Hoffman M, Bach FR, Blei DM Lafferty JD, Williams CKI, Shawe-Taylor J et al. Online learning for Latent Dirichlet Allocation. Advances in Neural Information Processing Systems 23. 2010; Colorado USA Curran Associates, Inc 856–64. 10.5555/2997189.2997285.

[43] Zhang B, Horvath S A general framework for weighted gene co-expression network analysis. Stat Appl Genet Mol Biol. 2005; 4:17 10.2202/1544-6115.1128. 16646834

[44] Aldinucci M, Bagnasco S, Lusso S et al. OCCAM: a flexible, multi-purpose and extendable HPC cluster. J Phys: Conf Ser. 2017; 898:82039.

[45] Rosenberg A, Hirschberg J V-measure: a conditional entropy-based external cluster evaluation measure. Proceedings of the 2007 Joint Conference on Empirical Methods in Natural Language Processing and Computational Natural Language Learning. 2007; Prague, Czech Republic 410–20.

[46] Shi H, Gerlach M, Diersen I et al. Chaudhuri K, Sugiyama M A new evaluation framework for topic modeling algorithms based on synthetic corpora. Proceedings of Machine Learning Research. Vol. 89, of Proceedings of Machine Learning Research. 2019; PMLR 816–26.

[47] Wolf FA, Angerer P, Theis FJ SCANPY: large-scale single-cell gene expression data analysis. Genome Biol. 2018; 19:15 10.1186/s13059-017-1382-0. 29409532 PMC5802054

[48] Satija R, Farrell JA, Gennert D et al. Spatial reconstruction of single-cell gene expression data. Nat Biotechnol. 2015; 33:495–502. 25867923 10.1038/nbt.3192 PMC4430369

[49] Eisenberg E, Levanon EY Human housekeeping genes, revisited. Trends Genet. 2013; 29:569–74. 10.1016/j.tig.2013.05.010. 23810203

[50] Zhou W, Yao S, Liu L et al. An overview of topic modeling and its current applications in bioinformatics. SpringerPlus. 2016; 5:1608 10.1186/s40064-016-3252-8. 27652181 PMC5028368

[51] Subramanian A, Tamayo P, Mootha VK et al. Gene Set Enrichment Analysis: a knowledge-based approach for interpreting genome-wide expression profiles. Proc Natl Acad Sci. 2005; 102:15545–50. 10.1073/pnas.0506580102. 16199517 PMC1239896

[52] Wang Q, Armenia J, Zhang C et al. Unifying cancer and normal RNA sequencing data from different sources. Scientific Data. 2018; 5:180061 10.1038/sdata.2018.61. 29664468 PMC5903355

[53] Wang Q, Gao J, Schultz N Unified RNA-seq datasets in human cancers and normal tissues - normalized data. Figshare. 2017; 2:5330593 10.6084/m9.figshare.5330593.v2.

[54] Grossman RL, Heath AP, Ferretti V et al. Toward a shared vision for cancer genomic data. New Engl J Med. 2016; 375:1109–12. 27653561 10.1056/NEJMp1607591 PMC6309165

[55] Chollet F et al. Keras. 2015; *https://keras.io*(9 April 2025, date last accessed).

[56] Melé M, Ferreira PG, Reverter F et al. The human transcriptome across tissues and individuals. Science. 2015; 348:660–5. 10.1126/science.aaa0355. 25954002 PMC4547472

[57] Furusawa C, Kaneko K Zipf’s Law in gene expression. Phys Rev Lett. 2003; 90:88102 10.1103/PhysRevLett.90.088102. 12633463

[58] Stoeger T, Gerlach M, Morimoto RI et al. Large-scale investigation of the reasons why potentially important genes are ignored. PLoS Biol. 2018; 16:e2006643. 30226837 10.1371/journal.pbio.2006643 PMC6143198

[59] Ghasemian A, Hosseinmardi H, Clauset A Evaluating overfit and underfit in models of network community structure. IEEE Trans Knowl Data Eng. 2020; 32:1722–35. 10.1109/TKDE.2019.2911585.

[60] Prosperi M, Guo Y, Sperrin M et al. Causal inference and counterfactual prediction in machine learning for actionable healthcare. Nat Mach Intell. 2020; 2:369–75. 10.1038/s42256-020-0197-y.

[61] Way GP, Zietz M, Rubinetti V et al. Compressing gene expression data using multiple latent space dimensionalities learns complementary biological representations. Genome Biol. 2020; 21:109 10.1186/s13059-020-02021-3. 32393369 PMC7212571

[62] Taylor MV, Hughes SM Mef2 and the skeletal muscle differentiation program. Semin Cell Dev Biol. 2017; 72:33–44. 10.1016/j.semcdb.2017.11.020. 29154822

[63] Owens GK, Kumar MS, Wamhoff BR Molecular regulation of vascular smooth muscle cell differentiation in development and disease. Physiol Rev. 2004; 84:767–801. 10.1152/physrev.00041.2003. 15269336

[64] Xu Y, Zhang H, Chen Y et al. SRF SUMOylation modulates smooth muscle phenotypic switch and vascular remodeling. Nat Commun. 2024; 15:6919 10.1038/s41467-024-51350-5. 39134547 PMC11319592

[65] Stuart T, Butler A, Hoffman P et al. Comprehensive integration of single-cell data. Cell. 2019; 177:1888–902. 10.1016/j.cell.2019.05.031. 31178118 PMC6687398

[66] Sarkar A, Stephens M Separating measurement and expression models clarifies confusion in single-cell RNA sequencing analysis. Nat Genet. 2021; 53:770–7. 10.1038/s41588-021-00873-4. 34031584 PMC8370014

[67] Booeshaghi AS, Pachter L Normalization of single-cell RNA-seq counts by log(x+1) or log(1+x). Bioinformatics. 2021; 37:2223–4. 10.1093/bioinformatics/btab085. 33676365 PMC7989636

[68] Han X, Wang R, Zhou Y et al. Mapping the mouse cell atlas by microwell-seq. Cell. 2018; 172:1091–107. 29474909 10.1016/j.cell.2018.02.001

[69] Bengio Y, Goodfellow I, Courville A Deep learning, vol. 1. 2017; Cambridge, MA, USA MIT Press.

[70] Peixoto TP Merge-split Markov chain Monte Carlo for community detection. Phys Rev E. 2020; 102:12305 10.1103/PhysRevE.102.012305. 32794904

[71] Yen TC, Larremore DB Community detection in bipartite networks with stochastic block models. Phys Rev E. 2020; 102:32309 10.1103/PhysRevE.102.032309. 33075933

[72] Peixoto TP Revealing consensus and dissensus between network partitions. Phys Rev X. 2021; 11:21003 10.1103/PhysRevX.11.021003.

[73] Valle F, Osella M, Caselle M topics. Zenodo. 2025; 10.5281/zenodo.15187437.

