# Supplementary material for: Exploring the latent space of transcriptomic data with topic modeling
Source: NAR Genom Bioinform. 2025 Apr 22;7(2):lqaf049. doi: 10.1093/nargab/lqaf049 (PMC12012681; doi:10.1093/nargab/lqaf049)
Supplement: lqaf049_Supplemental_File [file lqaf049_supplemental_file.pdf]

Exploring the gene expression latent space: a topic modeling approach

Filippo Valle[1,*], Michele Caselle[1] and Matteo Osella[1]

Normalised Mutual Information as evaluation metric

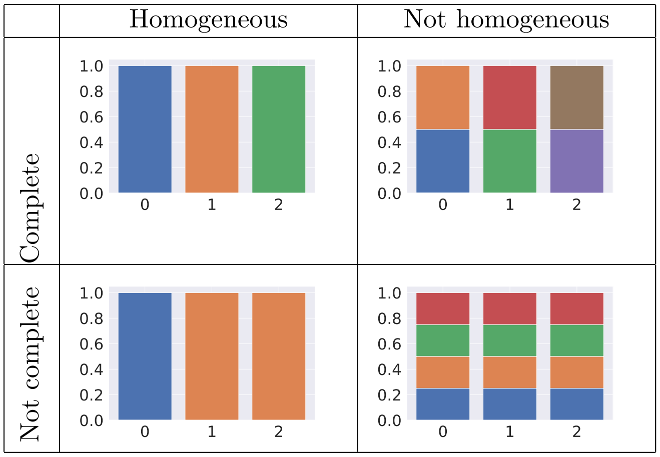

Table S1: Examples of homogeneity and completeness. Homogeneous clusters contain all nodes with the same label. A label is complete if it is fully represented by a single cluster. In this image, some examples of these definitions. The *NMI* score discussed in this work is nothing but the geometric average of completeness and homogeneity.

algorithms		NMI
hsbm	tm	0.256
hsbm	lda	0.070
hsbm	wgcna	0.058
tm	lda	0.150
tm	wgcna	0.656
lda	wgcna	0.147

Table S2: *NMI* between the genes mixtures in different algorithms (hierarchical doesn't provide information on genes).

algorithms		NMI
hsbm	tm	0.573
hsbm	lda	0.806
hsbm	wgcna	0.772
hsbm	hierarchical	0.867
tm	lda	0.659
tm	wgcna	0.707
tm	hierarchical	0.610
lda	wgcna	0.836
lda	hierarchical	0.852
wgcna	hierarchical	0.813

Table S3: *NMI* between the clusters mixtures in different algorithms.

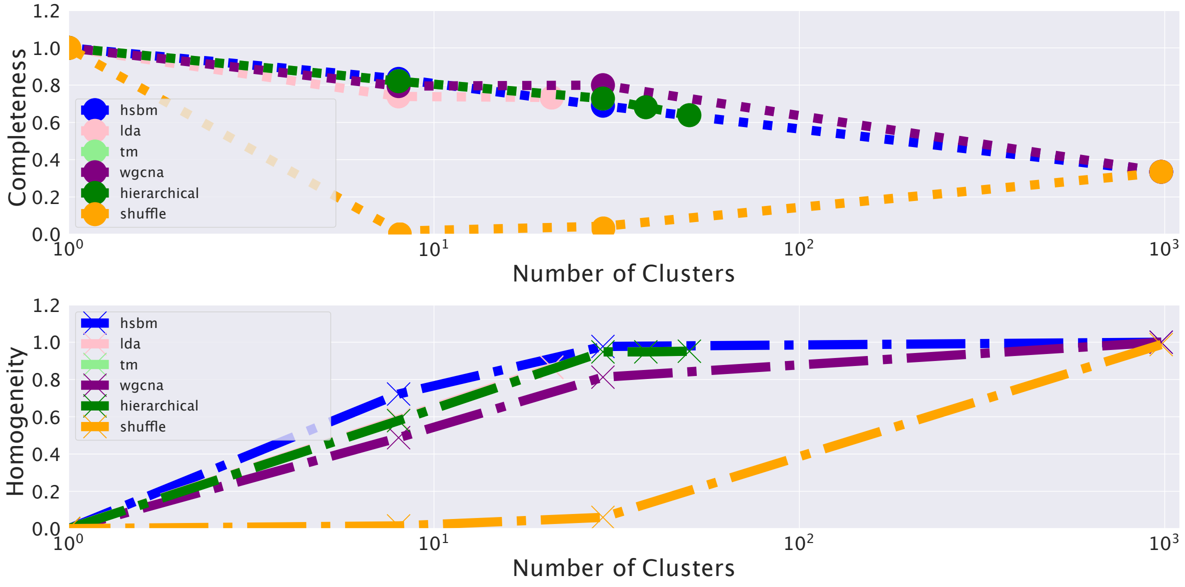

Figure S1: **Normalised mutual information as the geometric average of homogeneity and completeness.** As shown in Table S1 the Normalised Mutual Information (NMI), widely used in the main text, can be disentangled into homogeneity and completeness. In this Figure, we report the two different scores for the situation with 10 tissues for different algorithms. This should help to clarify the biological implication of this metric, as discussed in the main text. We would like to raise the attention of the reader on the fact that the first point (the one with all the samples in the same cluster) represents a trivial situation in which the homogeneity $h = 0$ and the completeness is $c = 1$ by definition.

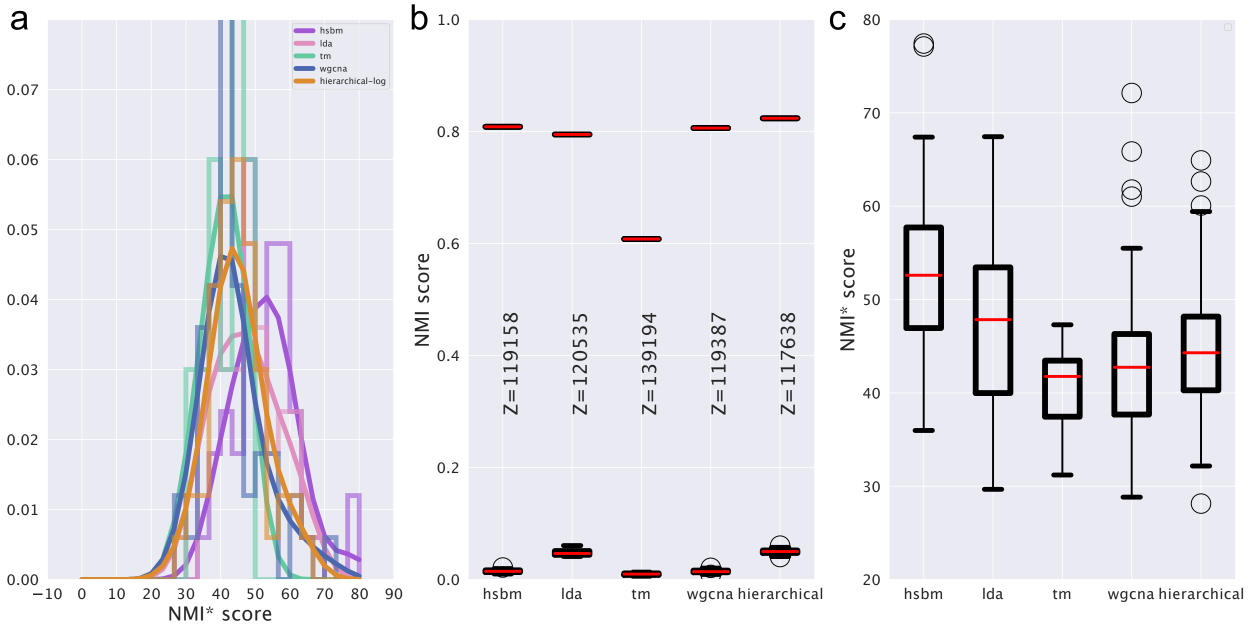

Figure S2: **NMI* score distributions for tissue classification problem in GTEx.** We estimated the NMI* using 50 different random partitions per each algorithm. **(a)** The distributions of these scores are approximately Gaussian distributed and indeed can be well fitted with Gaussian kernel (continuous lines). **(b)** The NMI scores (not divided by random) are quite distant from the NMI of a random partition. In this picture, we represented only the maximum reached in the hierarchy. In **(c)** we reported the NMI* score for each algorithm.

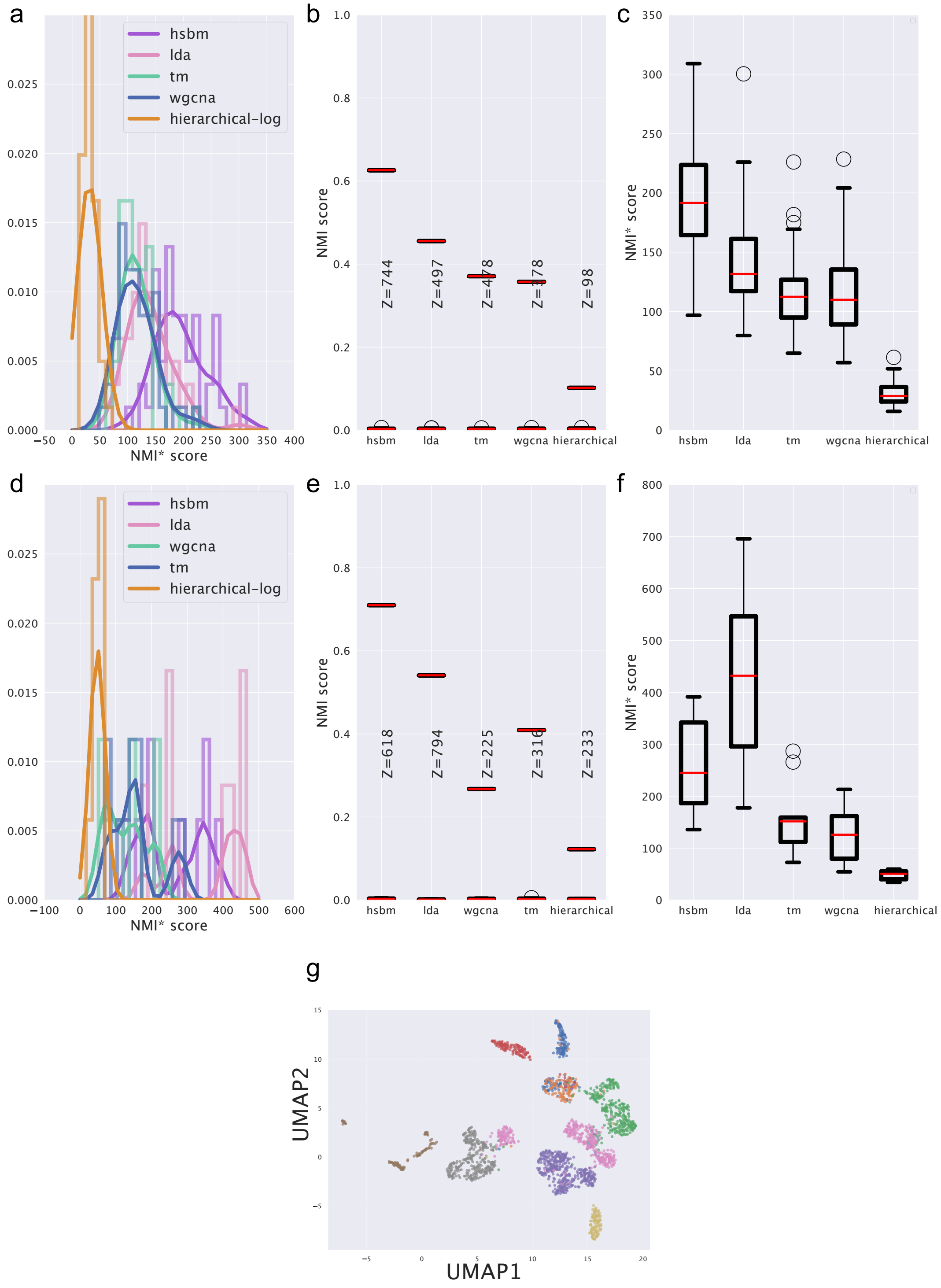

Figure S3: **NMI* score distributions** as in Figure S2, but in the single-cell settings. We estimated the NMI* multiple times with different random partitions per each algorithm. **(a)** The distributions of these scores are Gaussian distributed. The continuous lines are a Gaussian kernel. **(b)** The NMI scores (not divided by random) are quite distant from the NMI of a random partition. In this picture, we represented only the maximum reached in the hierarchy. In **(c)** the NMI* score for each algorithm. In **(d)**, **(e)** and **(f)** we report the same analysis but we considered the Cell type as the true label to estimate the scores. In this case, the number of labels is 20 (we choose 5 cell types per each of the 4 organs). Note that in this setting LDA found 4 clusters, hSBM 9, tm 7, WGCNA 5, and hierarchical 4, their null models could be a little different (they are estimated shuffling a different number of clusters). This is the reason for the slight differences in trends of **(e)** and **(f)**. In **(g)** we report a UMAP with cells highlighted depending on their cluster assigment by hSBM.

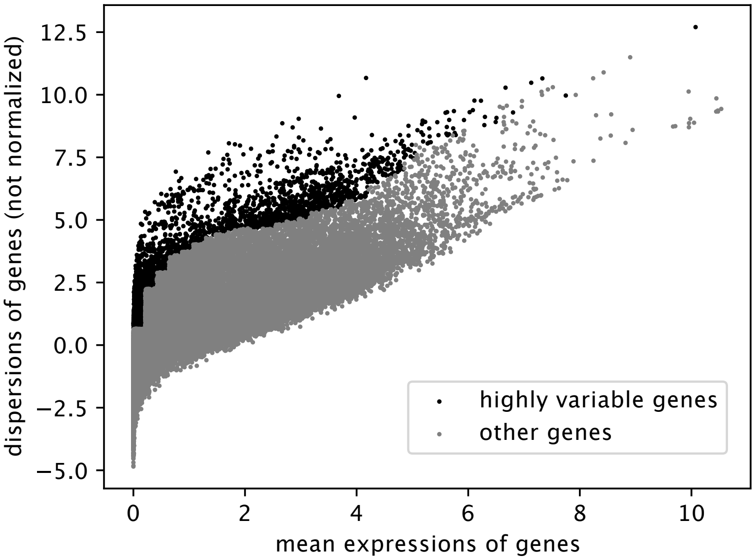

Figure S4: We use *scanpy* standard tool to select the highly variable genes. The dispersion is defined as $\frac{\sigma^2}{\text{mean}}$ and it scales with mean. Highly variable genes are the most dispersed in each bin of the mean. We used `scanpy.pp.highly_variable_genes(adata, n_top_genes = 3000, n_bins = 50)`

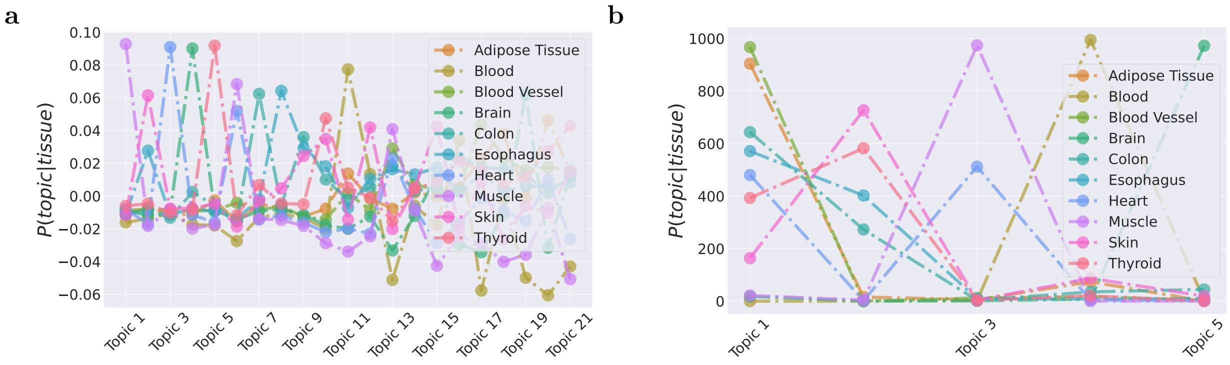

Figure S5: We reported $P(\text{topic}|\text{tissue})$ ($P(\text{topic}|\text{sample})$ averaged over tissues) for different tissues using the projections provided by (a) WGCNA and (b) Topic Mapping.

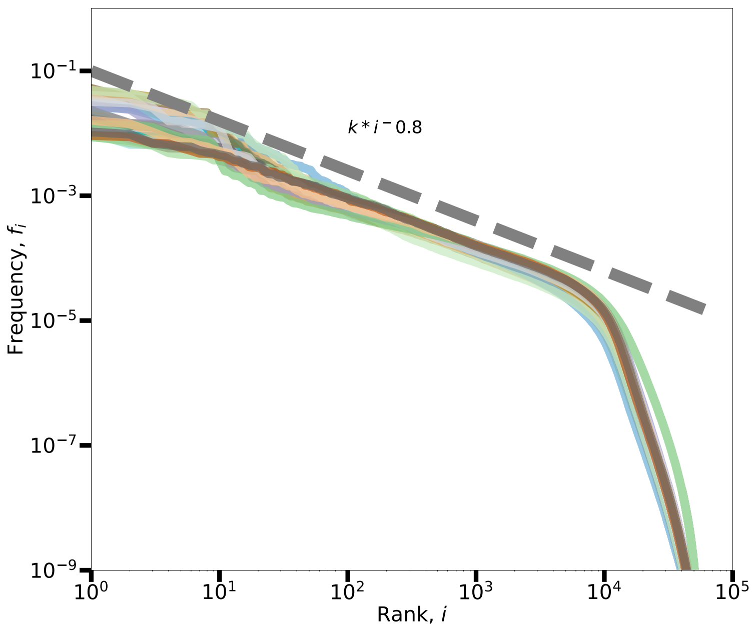

Figure S6: Rank plot of genes abundances in GTEx. The frequency f_i is estimated as $f_i = \frac{\Sigma_{s=1...R} n_{gs}}{\Sigma_{s=1...R} M_s}$ being $M_s = \Sigma_{g=1...N} n_{gs}$, R the number of samples and N the number of genes.

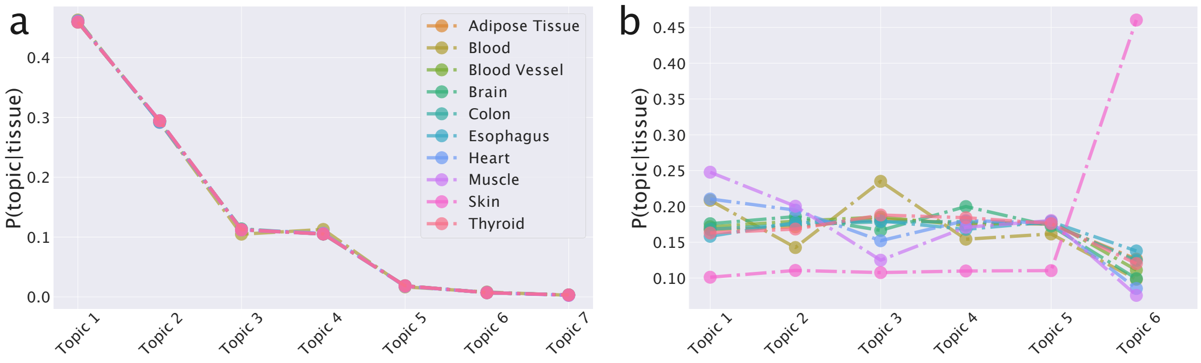

Figure S7: We reported $P(\text{topic}|\text{tissue})$ for different tissues for (a) hSBM and (b) LDA in experiments considering only housekeeping genes.

Biological insights from topics

The Figure S8 reports the values of the centered $P(\text{topic}|\text{sample})$ for one of the Topics at level three (Topic 6) which contains 75 genes. Looking at the Figure, we see that the topic is particularly associated to Blood, Heart, and Muscle. Looking at the gene content we find a strong enrichment of targets of three Transcription Factors (TF), using the usual enrichment score corrected for multiple testing using Gene Set Enrichment Analysis (GSEA) tool, we find the following FDR-values: *MEF2* $10^{-}11$, *AP4* $10^{-}13$ and *SRF* $10^{-}9$. In agreement with the tissue annotation, the functional annotation of this topic shows a strong enrichment (FDR-value=$10^{-}40$) for myogenesis. While the role of *MEF2* and *SRF* in Heart, Blood and Muscle development is rather well known (see the main text for literature references), the involvement of *AP4* is not known in the literature, but our analysis strongly suggests it. At level 2 (which is a finer representation of level 3 in the hierarchical organization) Topic 6 is split in several topics. We show the tissue association of three of them in Figure S8b,c,d. We see that the tissue annotation of Topic 6 is split at this level: Topic 2 and Topic 254 are strongly associated to Blood, while Topic 39 and 40 are associated to Muscle and Heart, but with a different ranking in the two cases. It is interesting that also the enrichment in targets of the three TFs splits at this hierarchical level: Topic 39 and 40 show enrichment for *MEF2* and *AP4*, but, again, with a different ranking: for Topic 39 we have a FDR for MEF2 $\sim 10^{-}4$ and AP4 $\sim 10^{-}6$; for Topic 40 we find *MEF2* $\sim 10^{-}10$ and *AP4* $\sim 10^{-}9$. At the same level Topic 2 and Topic 254 are enriched for *SRF* targets with an FDR-value of $\sim 10^{-}5$ and they are also enriched in functional annotations associated with Blood. These findings could help to better characterize the regulatory pathways driving the differentiation program of these tissues. Let us notice, as a final remark, that the splitting of Topic 6 at level 3 into different topics at level 2 is not a rigid association. Even if the majority of genes of Topic 6 group together genes from Topics 2, 39, 40 and 256 at the higher level, it also receives contributions from other topics at level 2. This mixed membership is the way in which the algorithm conveys the complexity of tissue organization at the gene level.

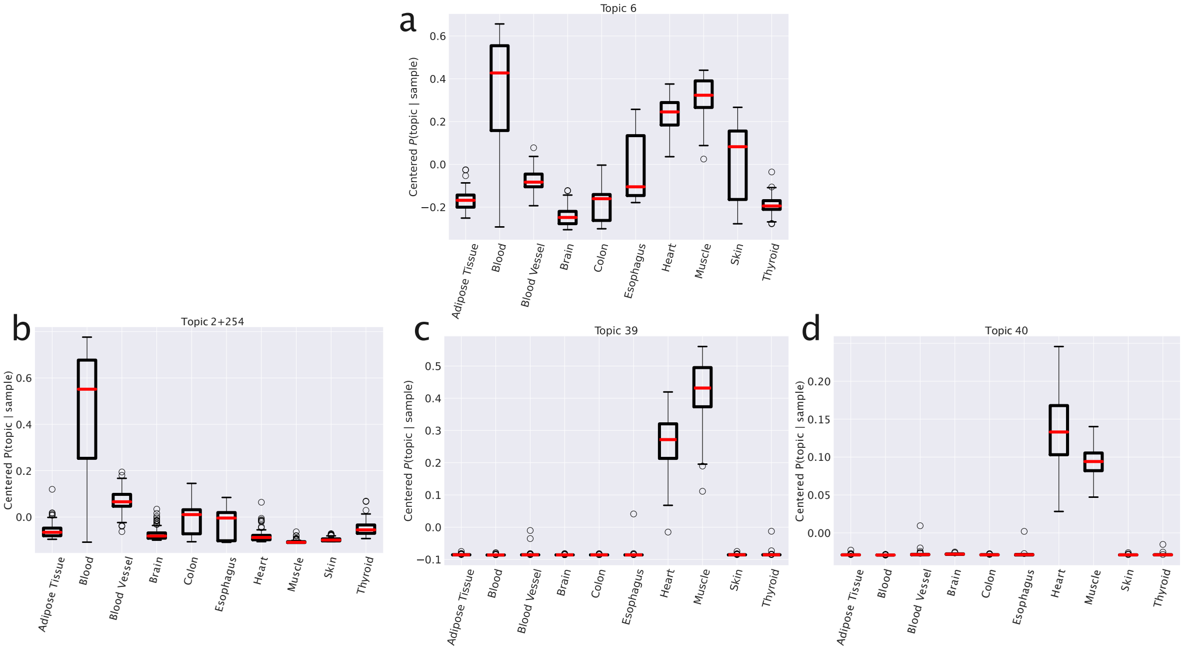

Figure S8: **In (a) we report Topic 6 at level 3 and in (b) Topics 2 and 259, (c) 39 and (d) 40 at level 2** of the hSBM hierarchy. These particular Topics were chosen because Topic 6 is the union of Topic 2, 39, 40 and 256 at the next hierarchy and they represent a playground to study the biological implications of the hierarchical structure of topics.

Significant Topics and their enrichment terms

We report in the following Figures and Tables some examples of significant topics for different algorithms. Next to the Figures with the $\bar{P}(\text{topic}|\text{tissue})$ we report the most enriched terms associated with the topics. As we discussed in the main text all the algorithms find topics associated with certain tissues, nevertheless the gene content of such topics is not consistent and this is reflected in the different terms found for different methods.

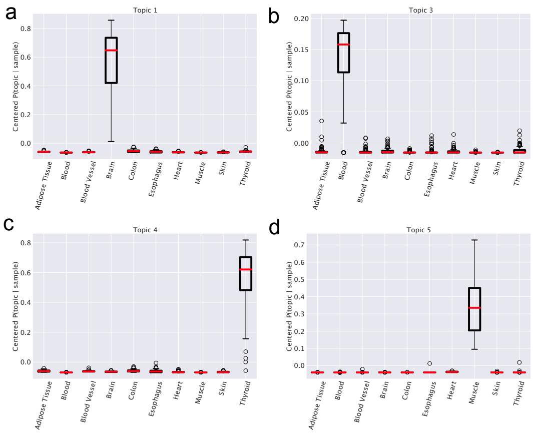

Term	FDR q-value
Topic 1 (20)	
GO_ANTIMICROBIAL_HUMORAL_RESPONSE	$6.44e-5$
CHARAFE_BREAST_CANCER _BASAL_VS_MESENCHNCHYMAL_UP	$6.44e-5$
Topic 3 (20)	
GSE22886_NAIVE_BCELL_VS_NEUTROPHIL_DN	$1.27e-14$
GSE29618_MONOCYTE_VS _PDC_DAY7_FLU_VACCACCINE_UP	$1.41e-12$
Topic 4 (20)	
GO_EPIDERMIS_DEVELOPMENT	$7.89e-23$
GO_SKIN_DEVELOPMENT	$5.85e-17$
Topic 5 (20)	
GSE22886_NAIVE_BCELL_VS_NEUTROPHIL_DN	$9.66e-12$
MODULE_84	$4.11e-8$

Figure S9: Gene ontologies enrichment test performed on different LDA's topics using GSEA. In brackets the number of genes.

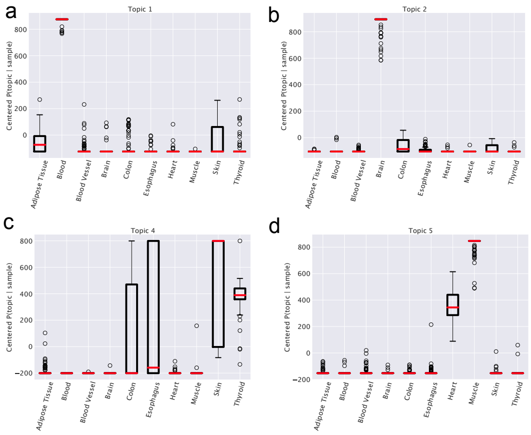

Term	FDR q-value
Topic 1 (26)	
CHEN_METABOLIC_SYNDROM_NETWORK	$1.92e-7$
RODWELL_AGING_KIDNEY_UP	$3.3e-7$
Topic 2 (24)	
MODULE_12	$1.79e-3$
GSE45365_NK_CELL_VS_CD8A_DC_DN	$1.79e-3$
Topic 4 (25)	
HOLLERN_EMT_BREAST_TUMOR_DN	$2.51e-22$
ONDER_CDH1_TARGETS_2_DN	$6.85e-19$
Topic 5 (23)	
GO_CONTRACTILE_FIBER	$1.4e-26$
GNF2_MYL2	$5.85e-19$

Figure S10: Gene ontologies enrichment test performed on different TM's topics using GSEA. In brackets the number of genes. TM outputs 5 topics, they are not enough to assign a topic to each tissue.

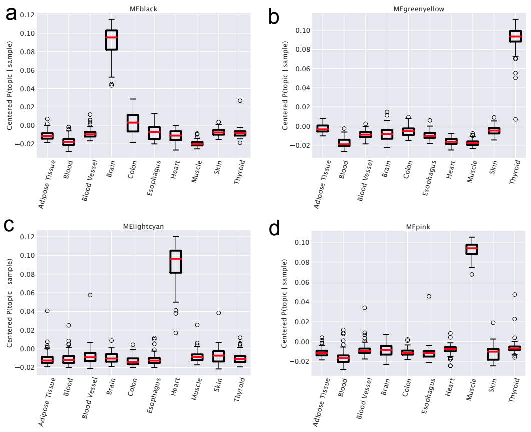

Term	FDR q-value
Topic black (389)	
GO_SYNAPSE	$4.64e-78$
GO_NEURON_PROJECTION	$1.19e-49$
Topic greenyellow (328)	
RODRIGUES_THYROID_CARCINOMA _POORLY_DIFDIFFERENTIATED_DN	$7.68e-27$
RODRIGUES_THYROID_CARCINOMA _ANAPLASTICTIC_DN	$1.2e-22$
Topic lightcyan (56)	
GO_CONTRACTILE_FIBER	$4.18e-31$
GO_MUSCLE_SYSTEM_PROCESS	$2.99e-26$
Topic pink (192)	
GO_CONTRACTILE_FIBER	$2.71e-66$
HALLMARK_MYOGENESIS	$8.52e-61$

Figure S11: Gene ontologies enrichment test performed on different WGCNA's topics using GSEA. In brackets the number of genes, in this setting the number of genes is hundreds, this can bias the hypergeometric P-value.

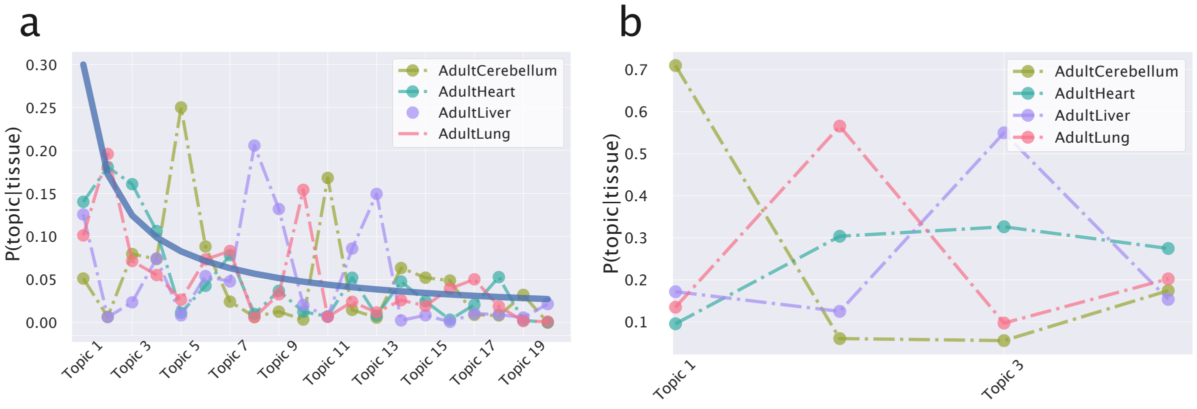

Figure S12: $P(\textbf{topic}|\textbf{tissue})$ **averaged for different organs in single-cells data.** The topic importance in samples of different tissues is reported in **(a)** for the hierarchical Stochastic Block Model and in **(b)** for LDA. Analogously to what it is shown in the main text for the bulk data hSBM tends to find a more global pattern meanwhile LDA tends to associate one single topic to each of the organs.

Predictor scores

In the paragraph about classifying samples with neural networks, we tested three settings:

- samples used to train topic modeling;
- samples projected in the topic space (these points are not used to train topic modeling);
- samples directly from the dataset without any processing;

All the results for a Neural Network NN and for k-NN are reported in the following tables.

data space	log-transformed data space	unseen data
	Primary site (tissue)	
acc: 0.8375 auc: 0.838	acc: 0.969, auc: 0.983	acc: 0.820 auc: 0.902
	Status (healthy or diseased)	
acc: 0.8375 auc: 0.8377	acc: 0.9875 auc: 0.9839	acc: 0.9226 auc: 0.9268
	All healthy primary sites	
acc: 0.9362 auc: 0.9659	acc: 0.9804 auc: 0.9896	n.a.

Table S4: Scores using the K-NN model. The rows represent the three different datasets discussed in the main text: 10 healthy tissues, unified healthy and diseased tissues, and all GTEx tissues. The scores in the first two columns are estimated in the original data space before and after applying the log-transformation. The last column represents samples not used for training topic modeling.

test set	unseen data
Primary site (tissue)	
acc: 1 auc: 1	acc: 0.9273 auc: 0.9852
Status (healthy or diseased)	
0.9750 auc: 1	acc: 0.9474 auc: 0.9762
All healthy primary sites	
acc: 0.9333 auc: 0.9980	n.a.

Table S5: Neural Network scores in different sets. The rows represent the three different datasets: 10 tissues, Healthy and diseased tissues, and all GTEx tissues. The first column represents the test error when using samples passed through topic modeling. The second column represents the test error when using points just projected into the topic space not being used for training the topic model.
